# Heme ligation and redox chemistry in two bacterial thiosulfate dehydrogenase (TsdA) enzymes

**DOI:** 10.1074/jbc.RA119.010084

**Published:** 2019-08-29

**Authors:** Leon P. Jenner, Julia M. Kurth, Sebastian van Helmont, Katarzyna P. Sokol, Erwin Reisner, Christiane Dahl, Justin M. Bradley, Julea N. Butt, Myles R. Cheesman

**Affiliations:** ‡ Centre for Molecular and Structural Biochemistry, School of Chemistry and School of Biological Sciences, University of East Anglia, Norwich Research Park, Norwich NR4 7TJ, United Kingdom; § Institut für Mikrobiologie & Biotechnologie, Rheinische Friedrich Wilhelms Universität Bonn, D-53115 Bonn, Germany; ¶ Department of Chemistry, University of Cambridge, Lensfield Road, Cambridge CB2 1EW, United Kingdom

**Keywords:** cytochrome, electron transfer, electrochemistry, ultraviolet-visible spectroscopy (UV-Vis spectroscopy), oxidation-reduction (redox), Campylobacter, biogeochemical sulfur cycle, magnetic circular dichroism (MCD), protein film electrochemistry (PFE), sulfur metabolism, tetrathionate reductase, thiosulfate oxidase, thiosulfate dehydrogenase

## Abstract

Thiosulfate dehydrogenases (TsdAs) are bidirectional bacterial di-heme enzymes that catalyze the interconversion of tetrathionate and thiosulfate at measurable rates in both directions. In contrast to our knowledge of TsdA activities, information on the redox properties in the absence of substrates is rather scant. To address this deficit, we combined magnetic CD (MCD) spectroscopy and protein film electrochemistry (PFE) in a study to resolve heme ligation and redox chemistry in two representative TsdAs. We examined the TsdAs from *Campylobacter jejuni*, a microaerobic human pathogen, and from the purple sulfur bacterium *Allochromatium vinosum*. In these organisms, the enzyme functions as a tetrathionate reductase and a thiosulfate oxidase, respectively. The active site Heme 1 in both enzymes has His/Cys ligation in the ferric and ferrous states and the midpoint potentials (*E_m_*) of the corresponding redox transformations are similar, −185 mV *versus* standard hydrogen electrode (SHE). However, fundamental differences are observed in the properties of the second, electron transferring, Heme 2. In *C. jejuni*, TsdA Heme 2 has His/Met ligation and an *E_m_* of +172 mV. In *A. vinosum* TsdA, Heme 2 reduction triggers a switch from His/Lys ligation (*E_m_*, −129 mV) to His/Met (*E_m_*, +266 mV), but the rates of interconversion are such that His/Lys ligation would be retained during turnover. In summary, our findings have unambiguously assigned *E_m_* values to defined axial ligand sets in TsdAs, specified the rates of Heme 2 ligand exchange in the *A. vinosum* enzyme, and provided information relevant to describing their catalytic mechanism(s).

## Introduction

Redox active molecules within the biogeochemical sulfur cycle can serve as sources of, or sinks for, the electrons transferred in energy-conserving pathways. An example is the oxidative coupling of two thiosulfate molecules to form tetrathionate seen in Equation 1 (1):

(Eq. 1) 2 S−-SO3−⇌ −O3S-S-S-SO3−+2e−(Em=+198 mV versus SHE)

This oxidation is well-established in many obligatory chemolithoautotrophic sulfur-oxidizing bacteria and also serves as a source of electrons for some purple (non)-sulfur bacteria (2). The reverse reaction, tetrathionate reduction, can terminate anaerobic respiratory electron transfer chains in a process thought to confer growth advantage in the microaerobic environment of intestinal mucosa (3). Both thiosulfate oxidation and tetrathionate reduction are catalyzed by the phylogenetically widespread family of periplasmic thiosulfate dehydrogenase (TsdA)^6^ enzymes (2).

**Figure 1.**
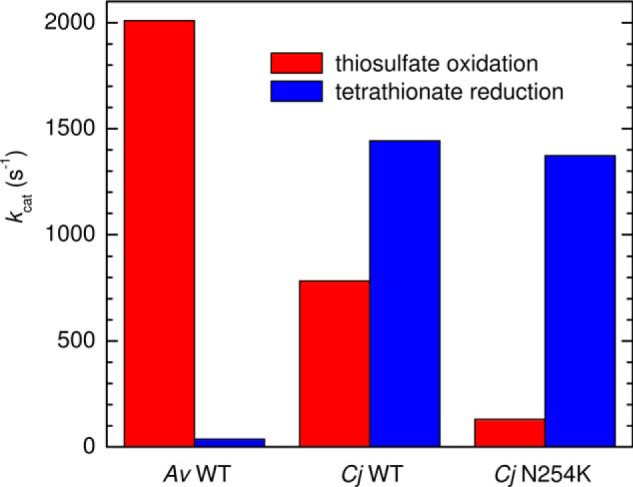
Maximum turnover numbers (*k*_cat_) for thiosulfate oxidation (*red*) and tetrathionate reduction (*blue*) by *Av* and *Cj* WT TsdA enzymes, and the *Cj* N254K variant, as determined by *in vitro* spectrophotometric enzyme activity assays using ferricyanide as an electron acceptor for thiosulfate oxidation and reduced methyl viologen as an electron donor for tetrathionate reductions. Adapted from Refs. 4 and 5.

Two of the best-characterized di-heme TsdA enzymes are those from the purple sulfur bacterium *Allochromatium vinosum* (*Av*) and the microaerobe *Campylobacter jejuni* (*Cj*), the latter a commensal in the small intestine of birds and one of the main causes of bacterial food-borne disease in humans. These enzymes have 41% sequence similarity (Fig. S1) and contain two *c*-type hemes. Nevertheless their cellular roles illustrate the contrasting activities required of TsdA: thiosulfate oxidation by *Av*TsdA provides electrons for photosynthesis whereas *Cj*TsdA allows use of tetrathionate as a terminal respiratory electron sink (3). However, spectrophotometric assays (4, 5) of the purified enzymes reveal detectable catalysis in both directions (Fig. 1), showing that neither is restricted to performing the physiologically required redox transformation. For *Cj*TsdA the maximum rate of tetrathionate reduction is approximately twice that of thiosulfate oxidation. For *Av*TsdA the maximum rate of oxidative catalysis greatly exceeds that of reductive catalysis. For TsdA enzymes isolated to date from other organisms (4–7) the relative rates of these two activities also correlate with their metabolic role, however, the molecular basis of these differing behaviors remains unclear.

**Figure 2.**
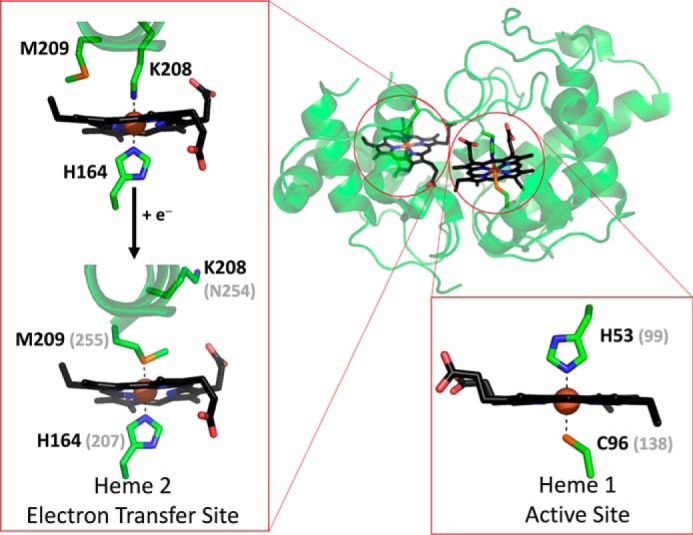
**The structure of *A. vinosum* TsdA with the two heme cofactors highlighted in *black*.** Expanded regions show the ligands to Heme 1 and those to Heme 2 in the oxidized (Fe(III)) and reduced (Fe(II)) states. Carbon atoms are shown in *green*, nitrogen in *blue*, oxygen in *red* and sulfur in *orange*. Where appropriate, *gray* values in parentheses indicate the corresponding residues from *C. jejuni* TsdA, as deduced from sequence alignment. Reproduced from PDB entries 4WQ7 and 4WQ9.

The crystal structure of as-isolated (di-Fe(III)) *Av*TsdA (4, 6) shows the closest edge-to-edge approach of the two hemes to be 8.1 Å, consistent with rapid interheme electron transfer. The proximal and distal axial ligands, respectively, are His^53^ and Cys^96^ to Heme 1 and His^164^ and Lys^208^ to Heme 2 (Fig. 2). However, crystals exposed to reducing agent (dithionite ion) show Lys^208^ is replaced by Met^209^ as distal ligand to Heme 2, and this exchange is a proposed consequence of a two-electron reduction within the crystal (4). Several observations point toward Heme 1 having an intimate role in thiosulfate/tetrathionate interconversion. The catalytic activity of *Av*TsdA in both directions is abolished by site-directed mutagenesis of Cys^96^ (4). Two nearby and conserved residues, Arg^109^ and Arg^119^ (see Fig. S1 for comparison of TsdA sequences), are important for activity and proposed to play a role in substrate binding by analogy with the SoxAX cytochromes that catalyze formation of a disulfide bond between thiosulfate and a cysteine of SoxYZ (6). Furthermore, exposure of *Av*TsdA to substrates leads to covalent modifications of Cys^96^, some of which carry an additional sulfur and may be relevant to catalysis (4, 6).

The structural properties of *Av*TsdA provide a basis for predicting the heme ligands in *Cj*TsdA (5). Significantly Lys^208^ is not conserved and the corresponding residue in *Cj*TsdA is the noncoordinating Asn^254^ (Fig. S1). However, the four remaining *Av*TsdA heme ligands, namely Cys^96^, Met^209^, and the two proximal histidines, are conserved in *Cj*TsdA (2). Thus, in all oxidation states of *Cj*TsdA the corresponding residues His^99^/Cys^138^ and His^207^/Met^255^ are implicated as the ligand sets to Heme 1 and 2, respectively (5). Replacing Cys^138^ with either His or Met produced proteins, *Cj*TsdA C138H and *Cj*TsdA C138M, respectively, that lack catalytic activity in both directions (5). The impact of possibly re-creating at *Cj*TsdA Heme 2 the His/Lys ligation of *Av*TsdA, and potentially a redox-linked ligand switch at that center, was tested by introducing lysine as residue 254 (5). The maximum rate of tetrathionate reduction by the resulting protein, *Cj*TsdA N254K, was comparable with that achieved by *Cj*TsdA but that of thiosulfate oxidation was ∼10-fold slower (Fig. 1). The catalytic behavior of the *Cj* enzyme did not become more like that of *Av*TsdA following modification to the Heme 2 environment.

In contrast to our knowledge of TsdA activities, information on their redox properties in the absence of substrates is lacking. In a previous report we described spectroelectrochemical characterization of *Av*TsdA adsorbed on SnO_2_ electrodes (8). Protein reduction was observed on poising the electrode at increasingly negative potentials, from +150 to −359 mV *versus* SHE. However, reoxidation was slower to occur and incomplete even at +330 mV. The complex behavior, combined with the lack of structural information afforded by electronic absorption spectroscopy, prevented unambiguous assignment of redox properties to Heme 1 or 2. As a consequence we were motivated to use a combination of magnetic circular dichroism (MCD) and protein film electrochemistry (PFE) to gain greater insight into the redox properties of TsdA enzymes. Here we present the corresponding studies of *Av*TsdA, *Cj*TsdA, and variants of the latter, namely *Cj*TsdA N254K, *Cj*TsdA C138H, and *Cj*TsdA C138M. *E_m_* values for Fe(III) ⇌ (II) transitions of TsdA hemes are defined, and the greater resolving power of MCD as compared with electronic absorbance spectroscopy allows, for the first time, their unambiguous assignment to sites of defined axial ligand sets. Furthermore, we provide insight into the rates of Heme 2 ligand exchange. The results address a significant gap in our knowledge of TsdA enzymes and contribute to wider discussions relating to the catalytic mechanism(s) operating in this enzyme family.

## Results

### MCD characterization of Av and Cj TsdA enzymes

MCD probes the same electronic transitions as electronic absorption spectroscopy but offers significant advantages. Signed bands and substantial variations in intensity of the porphyrin π-π* transitions in the UV-visible region identify, and quantify, the spin and oxidation state of the iron. Furthermore, at longer wavelengths, MCD can locate ligand to metal charge transfer (CT) transitions that are diagnostic of the axial ligands to Fe(III) heme (9, 10): a pair of bisignate bands (CT_1_ at 800–1300 nm and CT_2_ at 600–660 nm) for high-spin; a single positive band for each low-spin heme (CT_LS_ at 1000–2500 nm).

**Figure 3.**
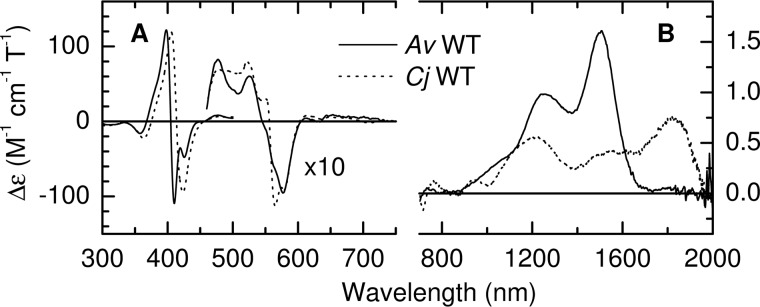
*A* and *B*, the MCD spectra of oxidized *A. vinosum* (*solid line*) and *C. jejuni* (*dashed line*) TsdA in the ranges (*A*) 300–750 nm and (*B*) 700-2000 nm. Protein concentrations were 34 μm (300–500 nm) and 180 μm (460–2000 nm) for *Av*TsdA and 17 μm (300–530 nm), 176 μm (460–750 nm) and 147 μm (700–2000 nm) for *Cj*TsdA. Data recorded at room temperature in 50 mm HEPES, 50 mm NaCl, pH 7, or the same buffer in D_2_O pH* 7 for nIR MCD.

MCD spectra of the fully oxidized TsdA enzymes are presented in Fig. 3. *Cj*TsdA displays two nIR CT_LS_ bands at 1215 nm and 1825 nm (Fig. 3*B*) with the latter having an associated vibrational sideband near 1600 nm (nIR MCD wavelengths for low-spin Fe(III) centers characterized in this work are collected in Table S1). The 1825-nm wavelength is diagnostic of His/Met ligated Fe(III) heme and accordingly is assigned to Heme 2. The 1215-nm band lies in the range of wavelengths reported for hemes coordinated by a nitrogen ligand distal to Cys^−^ (10–12) and is assigned to Heme 1 (the Cys^−^/H_2_O ligation found in P450 cytochromes results in an nIR CT band to shorter wavelength, 1050–1100 nm) (11). However, it should be noted that the nIR CT bands resulting from R-S-S^−^ (persulfide or sulfane) ligation may lie at similar wavelengths to those for cysteinate (13–15). Between 300 and 800 nm, the MCD is dominated by a bisignate feature at 415 nm characteristic of low-spin Fe(III) heme and with a peak to trough intensity of 215 m^−1^ cm^−1^ T^−1^ (Fig. 3*A*). For His/Cys^−^ ligated Fe(III) hemes, such features have an anomalously low peak to trough intensity of 50–90 m^−1^ cm^−1^ T^−1^, compared with ∼150 m^−1^ cm^−1^ T^−1^ typical for other ligand sets (16–25). The 415 nm intensity from *Cj*TsdA is therefore entirely consistent with His/Cys^−^ ligation at Heme 1 and His/Met ligation at Heme 2. MCD thus confirms, for *Cj*TsdA in solution, the heme ligands predicted from sequence alignments and electronic absorbance spectroscopy (5).

Interpretation of the MCD of oxidized *Av*TsdA (Fig. 3*B*) is more complex than for *Cj*TsdA. A positive CT feature is observed at 1505 nm, a wavelength diagnostic of low-spin Fe(III) heme with two nitrogenous axial ligands and therefore indicative of His/His or His/Lys ligation (10). However, the intensity is significantly higher than observed for any His/His ligated Fe(III) heme but similarly high intensities have been reported for examples of His/Lys(or amine) coordination (26–28). The His/Lys coordination, observed at Heme 2 in the crystallographically determined structure of *Av*TsdA, is therefore also present in the oxidized enzyme in solution. The feature at ∼1250 nm is unusually pronounced and unlikely to arise entirely from the vibrational sideband to the 1505 nm band. The more likely explanation is that a CT band from Heme 1 also contributes to this feature. This is supported by the interpretation of the UV-visible MCD (discussed below) that necessitates the presence of an nIR CT band from Cys^−^/His-ligated low-spin Heme 1.

The general intensities and band shapes of *Av*TsdA MCD in the 300–600 nm region (Fig. 3*A*) can be interpreted in the same manner as for the corresponding *Cj*TsdA features shown in the same panel. Specifically, the spectrum indicates the presence of two low-spin Fe(III) hemes, one of which has a Cys^−^ ligand because the bisignate feature at 407 nm has a peak to trough intensity of ∼230 m^−1^ cm^−1^ T^−1^. The negative component of this feature clearly resolves distinct contributions from the two differently ligated low-spin Fe(III) hemes, whereas for *Cj*TsdA the corresponding feature is a single broad lobe. The narrower features resolved for *Av*TsdA are again consistent with the presence of bis-nitrogenous ligation proposed for Heme 2 from the nIR MCD.

### MCD characterization of semireduced and fully reduced AvTsdA

**Figure 4.**
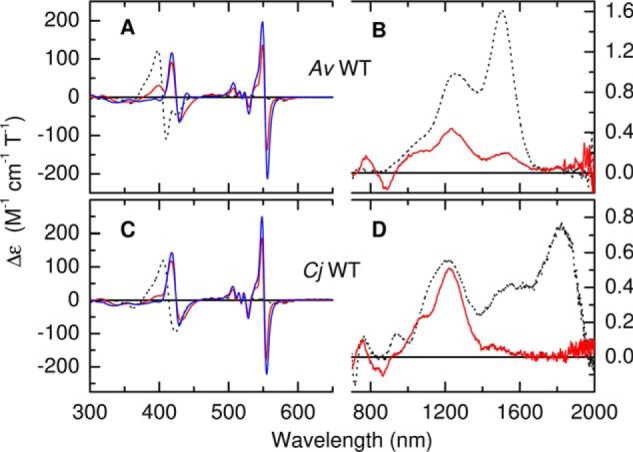
*A–D*, MCD spectra of *A. vinosum* (*A* and *B*) and *C. jejuni* (*C* and *D*) TsdA following incubation with sodium ascorbate (*red lines*) and sodium dithionite (*blue lines*). Protein concentrations were 35 and 20 μm (ascorbate and dithionite respectively, 300–700 nm) and 178 μm (700–2000 nm) for *Av*TsdA and 37 and 36 μm (300–700 nm) and 145 μm (700–2000 nm) for *Cj*TsdA. The *broken lines* are spectra of the fully oxidized enzymes from Fig. 3 for comparison. Data recorded at room temperature in 50 mm HEPES, 50 mm NaCl, pH 7, or the same buffer in D_2_O pH* 7 for nIR MCD.

**Scheme 1.**
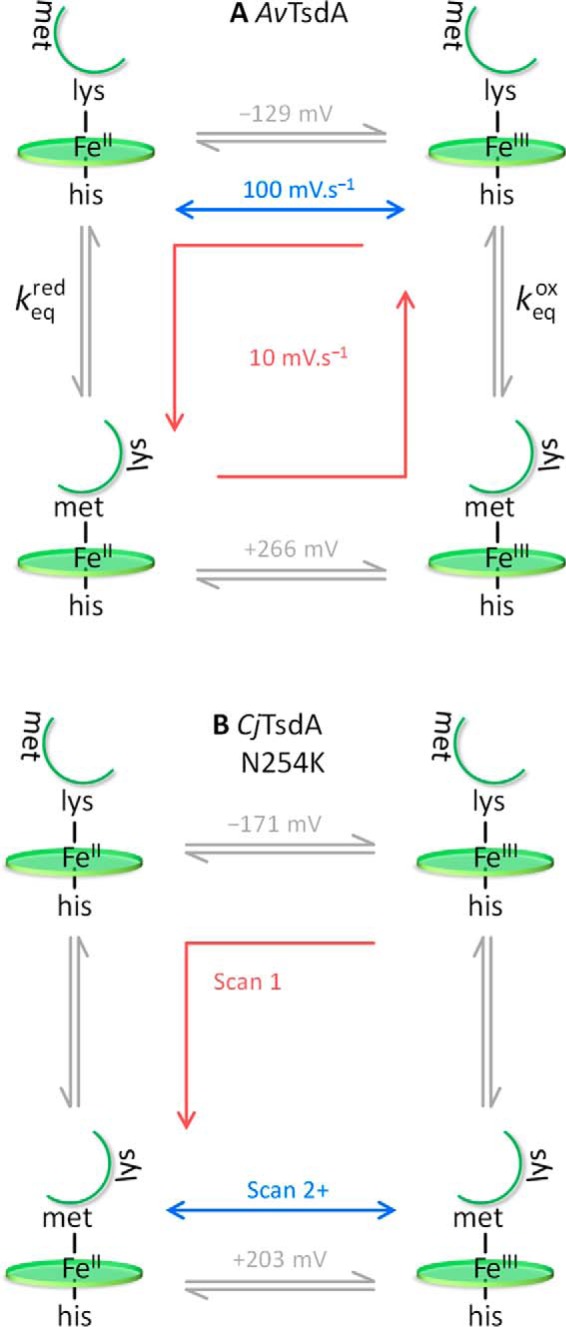
*A* and *B*, the redox-linked His/Met ⇌ His/Lys ligand switch at Heme 2 in (*A*) *Av*TsdA and (*B*) *Cj*TsdA N254K.

Fig. 4, *A* and *B* (*red*) shows the MCD of *Av*TsdA following incubation with 1.5 mm ascorbate (producing an effective potential of ≈60 mV; see “Experimental procedures”). There is a loss of intensity, compared with the MCD of the oxidized enzyme (*black dotted*), in the 407-nm bisignate feature associated with low-spin Fe(III) heme, together with the appearance of two features characteristic of low-spin Fe(II) heme, namely an asymmetric bisignate feature at 425 nm and a narrow bisignate band centered at 554 nm with a peak to trough intensity of ∼275 m^−1^ cm^−1^ T^−1^. The atypical MCD properties of Cys^−^ ligated hemes are such that, in the Fe(II) state, they give rise to broad and relatively weak visible region bands (17). The high intensity and narrow linewidth of the 554 nm feature suggests therefore that it could arise from reduction of Heme 2. This is confirmed by the nIR MCD (Fig. 4*B*) in which the intensity at 1505 nm because of His/Lys Fe(III) heme has diminished to ∼13% of that observed for fully oxidized *Av*TsdA. Concomitant loss of the corresponding vibrational sideband at wavelengths between 1100 and 1400 nm means that there should now be negligible MCD intensity in this region from Heme 2. However, significant intensity persists at these wavelengths and, as suggested earlier, can be assigned as the CT band of His/Cys^−^ ligated Fe(III) Heme 1, which is unaffected by ascorbate. The peak wavelength, at 1240 nm, matches that of the corresponding feature for the oxidized *Cj* enzyme (Fig. 3*B*). A peak to trough intensity at 554 nm of ∼275 m^−1^ cm^−1^ T^−1^ for 87% reduction of Heme 2 implies a value of ∼315 m^−1^ cm^−1^ T^−1^ for full reduction. This is lower than typically observed for hemes with bis-nitrogenous coordination but falls in the range observed for His/Met ligation, implying that a ligand switch has occurred on reduction of Heme 2, as illustrated in Scheme 1*A*.

Following anaerobic incubation of *Av*TsdA with the stronger reductant dithionite (1.5 mm, effective potential ≈ −500 mV), the MCD in the wavelength range 300–600 nm (Fig. 4*A*, *blue*) showed no features associated with Fe(III) heme, demonstrating complete reduction of both hemes. The dominant low-spin Fe(II) bisignate feature at 554 nm has increased in intensity compared with the ascorbate reduced form. However, the increase is almost four times that anticipated for reduction of the residual (∼13%) of Fe(III) His/Lys Heme 2, implying that reduced Heme 1 makes a significant contribution to this feature and one that is larger than expected for simple reduction of a His/Cys^−^ ligated heme. A similar observation was reported for the reduction of the active site His/Cys^−^ ligated heme in SoxAX and was attributed to protonation of Cys^−^ upon reduction (29). However, it should also be noted that a similar spectral response is anticipated if Cys^96^ is replaced by hydrogen sulfide as was observed in the crystal structure following dithionite reduction. A small positive band at 440 nm, characteristic of high-spin Fe(II) heme and with an intensity (12 m^−1^ cm^−1^ T^−1^) sufficient to account for 0.10–0.25 of a heme, is also consistent with the crystal structure that showed ∼25% of Heme 1 in a five-coordinate state.

### MCD characterization of semireduced and fully reduced CjTsdA

Incubation of *Cj*TsdA with 1.5 mm ascorbate also produces a semireduced form of the enzyme (Fig. 4, *C* and *D*, *red*). The resultant changes in the MCD spectrum are broadly similar to those described for *Av*TsdA. However, with negligible positive intensity at 400 nm and a peak to trough intensity at 550 nm of 365 m^−1^ cm^−1^ T^−1^ the extent of heme reduction by ascorbate is greater in *Cj* than *Av*TsdA. Indeed, in the nIR, all intensity above 1500 nm is removed, whereas that of the peak at 1210–1220 nm is unaffected. Therefore Heme 1 remains fully oxidized upon incubation of *Cj*TsdA with ascorbate and Heme 2 is fully reduced. This indicates a higher *E_m_* for Heme 2 in *Cj*TsdA than in *Av*TsdA, possibly because both oxidative and reductive transitions in the former involve only His/Met ligated heme. The peak to trough intensity of ∼365 m^−1^ cm^−1^ T^−1^ for the 554 nm feature is consistent with one fully reduced heme and comparable for that deduced for His/Met Heme 2 in *Av*TsdA.

Anerobic incubation of *Cj*TsdA with dithionite eliminates the features associated with Fe(III) Heme 1 from the MCD spectrum (Fig. 4*C*, *blue*) but produces an increase of ∼30% in the peak to trough intensity of the 550 nm feature, implying that, as with *Av*TsdA, protonation of the Heme 1 Cys^−^ accompanies reduction. In contrast to *Av*TsdA, the MCD of fully reduced *Cj*TsdA contains no intensity at 440 nm that might indicate the presence of high-spin Fe(II) heme.

### Protein film voltammetry of Av and Cj TsdA enzymes

**Figure 5.**
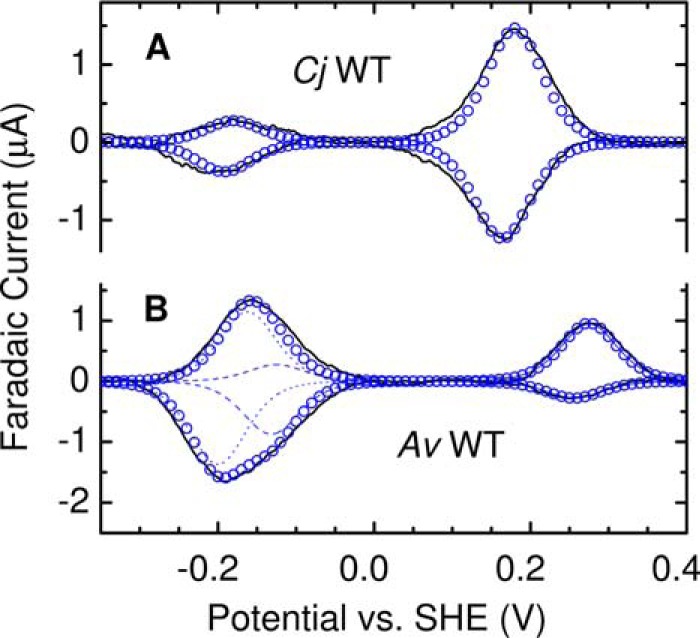
**Representative protein film cyclic voltammograms for *Cj*TsdA and *Av*TsdA as indicted.** Experimental data (*black solid lines*) for 10 mV s^−1^ scan rate in 50 mm HEPES, 50 mm NaCl, pH 7, 4 °C. For *Cj*TsdA, the summation of two modeled contributions at *E_m_* −186 mV and +172 mV is shown (*blue circles*). For *Av*TsdA, the summation (*blue circles*) comprises two modeled contributions from Heme 2 with *E_m_* values of +266 mV, −129 mV (*blue dashed lines*), and one from Heme 1 at −181 mV (*dotted blue lines*).

Insight into the redox properties associated with the fully equilibrated TsdA proteins is provided by the MCD described above. The redox activities fall in two well-separated regions, conveniently dissected by the potential of approximately +60 mV produced in solution by a 1.5-mm concentration of ascorbate (see “Experimental procedures”). In both proteins, Heme 1 remains fully oxidized in the presence of ascorbate but is fully reduced by dithionite, implying *E_m_* for the Fe(III)/Fe(II) couple lies in the approximate range −380 to −60 mV. Ascorbate fully reduces Heme 2 in *Cj*TsdA and >80% in *Av*TsdA, placing the apparent *E_m_* values above ≈ +100 mV in both proteins. Greater insight into the redox properties of both TsdA proteins was afforded by cyclic voltammetry after their adsorption on IO-ITO electrodes. These hierarchically structured electrodes (30) offer a large nanostructured surface area onto which the enzymes adsorb as electroactive films. Cyclic voltammetry reveals peaks (Fig. 5) corresponding to protein reduction (negative currents) and oxidation (positive currents). From these voltammograms, measured at a scan rate of 10 mV s^−1^, it is immediately apparent that the redox activities of both adsorbed proteins appear in two separated windows, one above ∼0 mV and the other between 0 and −350 mV, and so can be correlated with the behavior of the proteins in solution revealed by the MCD described above.

A (sub)monolayer film of an electroactive heme making an ideal, diffusionless contribution in cyclic voltammetry will give rise to a pair of peaks, one corresponding to oxidation and the other to reduction (31). These peaks would have equal areas as this property is proportional to the moles of electrons (*Q*) exchanged between heme and electrode. In addition, the peaks would ideally have the same peak potential, equal to *E_m_* of the corresponding Fe(III)/(II) couple, and at the temperature of our experiments (4 °C), the peaks would have half-height widths (δ) of ≈84 mV indicative of single-electron (*n* = 1) processes, rather than δ ≈42 mV if *n* = 2. Thus, for TsdA if both hemes give ideal contributions to cyclic voltammetry a superposition of two such responses is expected; two pairs of peaks, centered on separated *E_m_* values predicted by MCD, and having equal areas is expected. Our results show that neither TsdA displays cyclic voltammetry (Fig. 5) matching this prediction and that the response of *Cj*TsdA is clearly different from that of *Av*TsdA.

For *Cj*TsdA the voltammogram (Fig. 5*A*, *black*) contains four peaks each having δ of 80–100 mV, consistent with their origins in *n* = 1 transformations (Fig. 5*A*, *blue circles*). The two higher potential peaks have equal area and describe reversible reduction of a site with *E_m_* ≈ +172 mV (*E_m_* values are collected in Table S2). Given the MCD results, these peaks are attributed to ascorbate-reducible His/Met Heme 2. The two lower potential peaks are also of equal area. These describe a site with *E_m_* ≈ −186 mV and are attributed to dithionite reducible His/Cys^−^ Heme 1. The notable and unexpected observation is that transfer of ≈42 pmol *e*^−^ is determined by integration of the low potential oxidation peak (*Q*_lo_^ox^), whereas ≈166 pmol *e*^−^ are transferred in the high potential oxidation peak (*Q*_hi_^ox^**)**. Similar behavior was noted for the reductive peaks, for which *Q*_lo_^red^ ≈ 35% *Q*_hi_^red^. These properties were retained over multiple cycles and using scan rates from 5 to 100 mV s^−1^ (Fig. S3) for which there was negligible change in the total moles of electrons (*Q*_tot_) exchanged in the oxidative and reductive sweeps (integrated peak areas for measured and modeled contributions to the voltammograms of all proteins are summarized in Table S3). The simplest interpretation for these observations, and one that we return to consider below, is that significantly more His/Met ligated Heme 2 than His/Cys^−^ ligated Heme 1 is electroactive in the films of *Cj*TsdA.

At 10 mV s^−1^ the voltammetry of *Av*TsdA also resolves four peaks (Fig. 5*B*, *black*) and *Q*_tot_^ox^ ≈ *Q*_tot_^red^ (Fig. S3 and Table S3). There is again a disparity between the charge transferred in the peaks at high and at low potentials. However, the behavior differs from that of *Cj*TsdA in several respects. At high potentials the oxidative peak displayed by *Av*TsdA is larger than the corresponding reductive peak (*Q*_hi_^ox^ > *Q*_hi_^red^) and at low potentials the reductive peak is larger than the corresponding oxidative feature (*Q*_lo_^red^ > *Q*_hi_^ox^). Furthermore, although the peaks at high potential have δ ≈ 80–100 mV, indicative of *n* ≈ 1, those at low potential are significantly broader (δ ≈ 150 mV) and each shows structure suggesting two overlapping signals. Indeed, both low potential peaks could be reasonably described by the sum (Fig. 5*B*, *blue open circles*) of two contributions with δ = 84 mV having different *E_m_* values and areas (Fig. 5*B*, *dotted and dashed lines*). This behavior was reproduced in consecutive voltammograms.

In view of the MCD, the following interpretation of the *Av*TsdA voltammetry at 10 mV s^−1^ is proposed. The main contribution to the peaks at low potential (Fig. 5*B*, *blue dotted lines*) is assigned to reversible reduction of His/Cys^−^ Heme 1 with *E_m_* ≈ −181 mV. Other features in the voltammetry, at both high and low potentials, are assigned to Heme 2 for which electron transfer drives exchange of the distal ligand as described by anticlockwise progress around the states in the square scheme of Scheme 1*A*. At the most positive potentials of the voltammetry, *Av*TsdA is fully oxidized and Heme 2 has predominantly His/Lys ligation. On scanning to negative potentials, reduction of His/Lys Heme 2 occurs with *E_m_* ≈ −129 mV (Fig. 5*B*, *blue dashed line*). On returning to more positive potentials, the low potential oxidation peak is much smaller, representing oxidation of a remnant of His/Lys Heme 2, and a larger oxidation peak is resolved at ≈ +260 mV, revealing that the majority of Heme 2 is oxidized with His/Met ligation. Thus, it can be concluded that Met displaces Lys as the distal ligand of reduced Heme 2. During consecutive voltammograms the response is essentially the same as that of Fig. 5*B* so it can also be concluded that Lys displaces Met as the distal ligand to oxidized Heme 2 on the experimental timescale. In support of our proposal, summations of the deconvoluted features attributed to reduction and oxidation of Heme 2 describe transfer of similar numbers of electrons, ≈104 and ≈114 pmol *e*^−^ respectively. Furthermore, the features ascribed to redox transformation of Heme 1 account for transfer of ≈124 pmol *e*^−^. Thus, unlike for *Cj*TsdA, the electroactive populations of Heme 1 and Heme 2 are essentially equal for *Av*TsdA.

**Figure 6.**
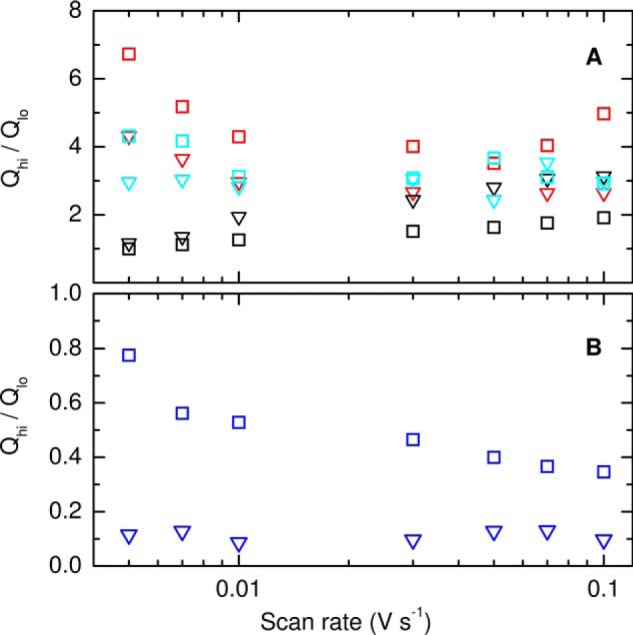
Ratio of moles of electrons transferred in high-potential (*Q*_hi_) and low-potential (*Q*_lo_) peaks for oxidation (*squares*) and reduction (*triangles*): *A*, *Cj*TsdA (*red*), *Cj*TsdA C138H (*black*), *Cj*TsdA N254K (*cyan*); *B*, *Av*TsdA (*blue*). Data points represent average values from four scans recorded in 50 mm HEPES, 50 mm NaCl, pH 7, 4 °C.

If the equilibrium for the *Av*TsdA Heme 2 ligand exchange is defined in the direction His/Lys ⇌ His/Met then *K*_eq_^red^/*K*_eq_^ox^, the ratio of the equilibrium constants in the two oxidation states, can be calculated from the reduction potentials in Scheme 1*A*: a ratio value of 5 × 10^6^ is consistent with the MCD results that showed the equilibrium lies significantly toward Lys coordination in the Fe(III) and Met coordination in the Fe(II) states of Heme 2. Some insight into the kinetics of this ligand exchange was afforded by the scan rate dependence of the *Av*TsdA voltammetric response (Fig. S4). At 100 mV s^−1^ a larger proportion of Heme 2 is oxidized with His/Lys ligation than at 10 mV s^−1^; the ratio *Q*_hi_^ox^/*Q*_lo_^ox^ (Fig. 6*B*, *squares* and Fig. S4 *circles*) is ≈35% smaller than at 10 mV s^−1^ although *Q*_hi_^red^/*Q*_lo_^red^ (Fig. 6*B*, *triangles*) and *Q*_tot_ (Fig. S4) are essentially unchanged. At the higher scan rate there is insufficient time for Met to fully displace Lys as the distal ligand to reduced Heme 2. Because Heme 1 contributes only to *Q*_lo_^ox^ and *Q*_lo_^red^, in the limiting case of full Lys → Met displacement at the reduced Fe(II) heme, *Q*_hi_^ox^/*Q*_lo_^ox^ → 1. In this study *Q*_hi_^ox^/*Q*_lo_^ox^ tends to 1 (Fig. 6*B*, *squares*) only at the lowest scan rate of 5 mV s^−1^ indicating that the Lys → Met switch at reduced Heme 2 occurs with a rate of ∼7 × 10^−2^ s^−1^. For the limiting case of full Met → Lys displacement at the oxidized Fe(III) Heme 2, *Q*_hi_^red^/*Q*_lo_^red^ → 0. In this study, *Q*_hi_^red^/*Q*_lo_^red^ is ∼0, even at the highest scan rates (Fig. 6*B*, *triangles*). This indicates the Met → Lys switch triggered by oxidation is always complete before re-reduction, which places a lower limit of ∼0.2 s^−1^ on the rate of this process.

The (limiting) ligand switching rates, when compared with the larger values of *k*_cat_ (Fig. 1), favor lysine over methionine during rapid turnover such that Heme 2 would remain His/Lys coordinated. The results of our previous spectroelectrochemical study of *Av*TsdA adsorbed on SnO_2_ electrodes (8) can be usefully considered in view of the present results. For that study the protein was poised at increasingly negative (+325 to −650 mV) and then increasingly positive potentials (−650 to + 325 mV) over a period of ∼2 h compared with the 6 min of the slowest cyclic voltammogram in this study. Changes in the electronic absorbance measured at the corresponding potentials suggested a fully reversible (Nernstian) redox transformation centered on approximately −225 mV and reduction between +150 and −100 mV of a second center for which reoxidation commenced above ∼+300 mV. We now confidently assign the reversible redox transformation to Heme 1 and the additional signals, evidencing hysteretic redox titration, to Heme 2, such that the observation of ligand switching at *Av*TsdA Heme 2 is independent of electrode material.

To investigate the origins of the different behaviors displayed by *Cj*TsdA and *Av*TsdA three previously prepared single-site variants of *Cj*TsdA (5) were studied as described below. First, we consider the properties of the *Cj*TsdA N254K variant produced to introduce key features of the *Av*TsdA Heme 2 distal pocket. Then, we consider properties of the *Cj*TsdA C138M and C138H proteins in which the Cys^−^ that ligates Heme 1 is replaced by Met and His, respectively.

### Spectroscopic and electrochemical characterization of CjTsdA N254K

As described above, distal ligation of *Av*TsdA Heme 2 is provided by Lys^208^ and Met^209^ (Fig. 2). The equivalent residues in *Cj*TsdA are Asn^254^ and Met^255^ (Fig. S1). MCD and protein film voltammetry of *Cj*TsdA N254K were performed to assess whether replacing Asn^254^ with Lys, *i.e.* creating an *Av*TsdA-like distal pocket, would introduce behavior characteristic of *Av*TsdA, namely, the Heme 2 ligand switching behavior Scheme 1*A*, and/or equal electroactive populations of Hemes 1 and 2.

**Figure 7.**
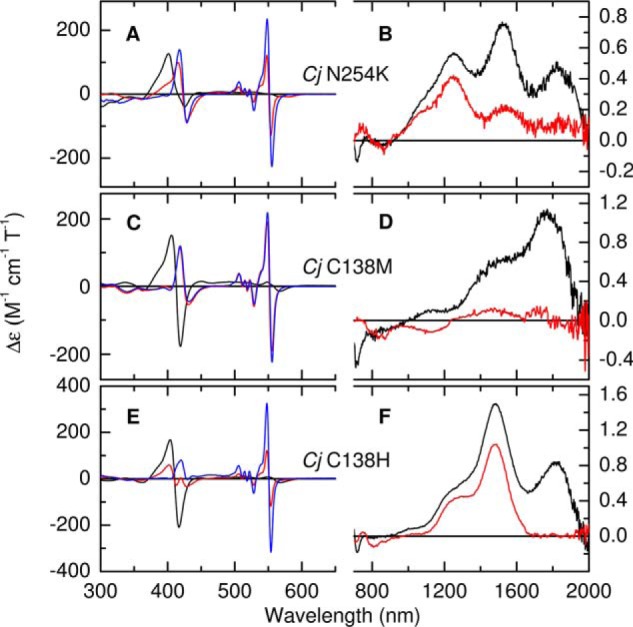
*A–F*, the MCD spectra of *Cj*TsdA variants N254K (*A* and *B*), C138M (*C* and *D*), and C138H (*E* and *F*), in the fully oxidized state (*black traces*), following incubation with sodium ascorbate (*red traces*) and with sodium dithionite (*blue traces*). Protein concentrations used for the 300–650 nm and the 700–2000 nm regions were, respectively, 47 μm and 325 μm; 40 μm and 160 μm; 18 μm and 169 μm. Data recorded at room temperature in 50 mm HEPES, 50 mm NaCl, pH 7, or the same buffer in D_2_O pH* 7 for nIR MCD.

For oxidized *Cj*TsdA N254K (Fig. 7*A*, *black*), the bisignate MCD feature at 413 nm is indicative of overlapping contributions from distinct Fe(III) hemes because it is asymmetric and broader than the corresponding features for *Cj*TsdA C138M and C138H. The spectrum lacks intensity at 600–700 nm, showing that neither heme has any high-spin component. The nIR MCD (Fig. 7*B*, *black*) is striking because it reveals CT bands for three distinct low-spin Fe(III) forms. The CT band of His/Cys^−^ coordinated Heme 1 is clearly resolved at 1250 nm (∼45 nm red shifted compared with *Cj*TsdA). The His/Met feature at 1825 nm has an intensity of 0.47 m^−1^ cm^−1^ T^−1^ showing that ∼64% of Heme 2 retains His/Met ligation. The prominent band at 1525 nm, not observed for *Cj*TsdA, indicates His/Lys coordination. Assuming that this accounts for the remaining ∼36% of Heme 2, and allowing for the underlying side-band from the His/Met form, suggests a normalized intensity for this His/Lys species of ∼1.4 m^−1^ cm^−1^ T^−1^. This high value is typical for His/Lys and comparable with that observed for Heme 2 in *Av*TsdA. Thus, introducing Lys in place of Asn^254^ in the *Cj*TsdA Heme 2-binding pocket allows Lys to provide the distal ligand of Heme 2 in a subpopulation of oxidized *Cj*TsdA N254K. The structure of the Heme 2 pocket, and not simply the amino acid residues, must exert a significant influence on the ligation at Heme 2 in *Cj*TsdA.

Incubation of *Cj*TsdA N254K with ascorbate (Fig. 7*A*, *red*) results in loss of MCD intensity in the 413 nm feature and the appearance of bisignate features at 422 and 551 nm corresponding to low-spin Fe(II) heme. The peak to trough intensity of the 551 nm feature is 250 m^−1^ cm^−1^ T^−1^, only 70% of that observed for ascorbate-treated *Cj*TsdA, suggesting incomplete reduction of Heme 2. This interpretation is confirmed by the observation of residual CT intensity at 1525 nm (Fig. 7*B*, *red*), sufficient to account for ∼15% of His/Lys Heme 2. Therefore, and in line with the behavior of *Cj*TsdA and *Av*TsdA, Lys/His-oxidized Heme 2 has a lower *E_m_* than His/Met Heme 2. Incubation with dithionite removes all features associated with Fe(III) heme from the nIR MCD and increases the intensities of the features at 422 and 551 nm (Fig. 7*A*, *blue*) in a manner consistent with complete reduction of Heme 1 and Heme 2.

**Figure 8.**
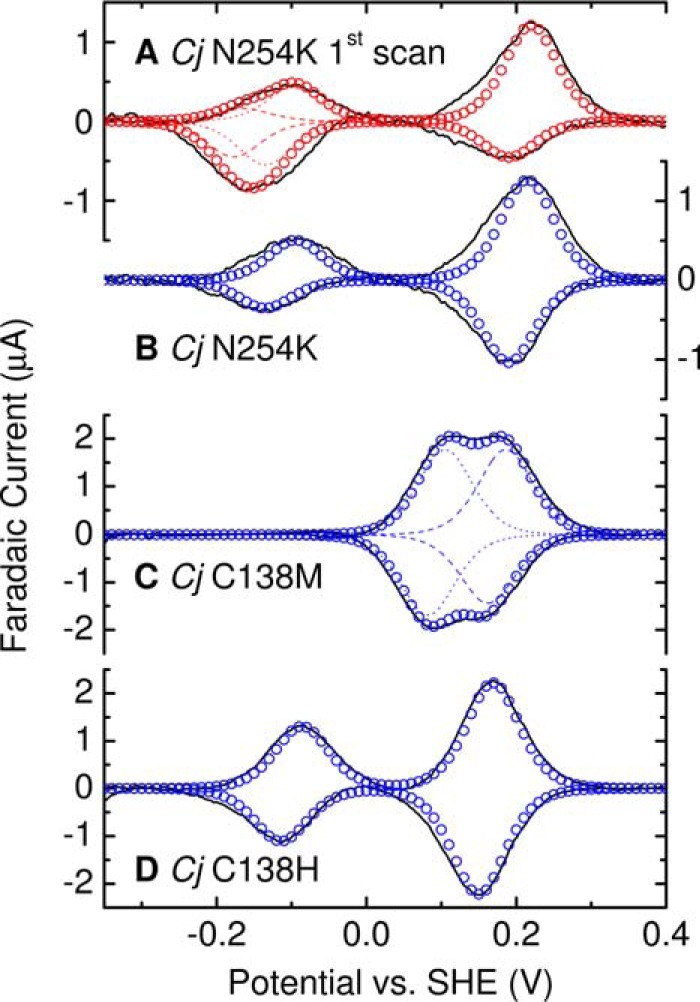
**Representative protein film cyclic voltammograms for adsorbed *Cj*TsdA N254K, C138M, and C138H, and as indicated.** Experimental data (*black solid lines*) for 10 mV s^−1^ scan rate in 50 mm HEPES, 50 mm NaCl, pH 7, 4 °C. For *Cj*TsdA N254K, first scan, summation (*open red circles*) of three modeled contributions with *E_m_* +203 mV and −115 mV (*red dotted lines*), −171 mV (*red dashed lines*). For *Cj*TsdA N254K, steady-state, summation (*open blue circles*) of two modeled contributions at *E_m_* −115 mV and +203 mV. For *Cj*TsdA C138M, summation (*open blue circles*) of two modeled contributions at *E_m_* +94 mV and +174 mV (*blue dashed lines*). For *Cj*TsdA C138H, summation (*open blue circles*) of two modeled contributions at *E_m_* −100 mV and +160 mV.

The voltammetry of *Cj*TsdA N254K at 10 mV s^−1^ (Fig. 8, *A* and *B*), as with the MCD spectra, was more complex than for the other proteins but its features can be assigned in light of the previously described behaviors. After initiating the voltammetry at +400 mV (Fig. 8*A*, *black*), a small reduction peak centered on ≈ +200 mV indicates reduction of His/Met Heme 2. Then a larger, broader peak between −50 and −300 mV comprises contributions from the reduction of His/Lys Heme 2 and His/Cys^−^ Heme 1 and can be modeled as the sum (*red circles*) of two *n* = 1 processes (*red lines*). The species reduced in this first scan therefore correspond to those identified by MCD. However, on returning to +400 mV the low potential oxidation peak is smaller than both the preceding low potential reduction peak *and* the subsequent high potential oxidation peak. The latter is larger than the peak describing the initial reduction of His/Met ligated Heme 2. These observations are reconciled if the population of His/Lys Heme 2 undergoes a Lys → Met ligand switch on reduction. Thus, on the first voltammetric cycle, the behavior of the His/Lys ligated population of Heme 2 in *Cj*TsdA N254K is strikingly similar to that in *Av*TsdA; on reduction the Lys ligand is displaced by Met (Scheme 1*B*).

The first voltammogram for *Cj*TsdA N254K at 10 mV s^−1^ (Fig. 8*A*, *black*) described above is clearly different from the second and subsequent voltammograms that are all similar and correspond to the steady-state for this scan rate (Fig. 8*B*, *black*). In the steady-state response, the high potential oxidation and reduction peaks have equal areas (*Q*_hi_^ox^ ≈ *Q*_hi_^red^) and are well-described by reversible transformation of an *n* = 1 center with *E_m_* ≈ +203 mV that we assign to His/Met ligated Heme 2. Smaller peaks at low potential correspond to reversible transformation of an *n* = 1 center with *E_m_* ≈ −115 mV that we assign to His/Cys^−^ Heme 1. Thus, on the timescale of these experiments in *Cj*TsdA N254K, Heme 2 becomes trapped with His/Met ligation in behavior that differs from *Av*TsdA. Applying the *E_m_* values deduced from the steady-state voltammetry to analysis of the first voltammogram yields *E_m_* ≈ −171 mV for the His/Lys Heme 2 (Fig. 8*A*, *red dashes*). With this assignment of the modeled contributions to the first voltammogram it is seen that approximately equal numbers of electrons are transferred to reduce the His/Met and His/Lys ligated populations of Heme 2 (Table S3), in accord with their relative populations deduced by MCD of the oxidized protein.

In the 10 mV s^−1^ steady-state voltammograms of *Cj*TsdA N254K, the features assigned to oxidation (reduction) of Heme 2 describe transfer of ≈130 pmol *e*^−^ and those assigned to oxidation (reduction) of Heme 1 describe transfer of ≈40 pmol *e*^−^. These values were relatively unchanged for steady-state voltammograms recorded for scan rates between 5 and 100 mV s^−1^ (Fig. 6*A* and Fig. S4). Thus, introducing Lys into the *Cj*TsdA distal pocket is not sufficient to produce a voltammogram with features from equal electroactive populations of His/Cys^−^ ligated Heme 1 and Heme 2 having either His/Met or His/Lys ligation. As this behavior contrasts to that described for *Av*TsdA, we studied *Cj*TsdA C138M and C138H to assess the impact of replacing the Cys^−^ ligand to Heme 1 with alternate residues that could also provide heme ligation.

### Spectroscopic and electrochemical characterization of CjTsdA C138M

The Soret feature at 413 nm in the UV-visible MCD of oxidized *Cj*TsdA C138M (Fig. 7*C*, *black*) is narrower, more symmetrical, and of greater peak to trough intensity (∼330 m^−1^ cm^−1^ T^−1^) than the equivalent *Cj*TsdA feature. All these properties indicate the presence of two low-spin Fe(III) hemes that do not have thiolate ligation. The nIR MCD (Fig. 7*D*, *black*) appears to contain a single CT feature at ∼1770 nm, in the region diagnostic of His/Met ligation. However, the increased width and intensity (1.1 m^−1^ cm^−1^ T^−1^), as compared with the CT feature observed for Heme 2 in *Cj*TsdA, indicate two overlapping bands. The MCD thus shows unambiguously that both hemes have His/Met axial ligation in oxidized *Cj*TsdA C138M and that Met^138^ is an axial ligand to Heme 1. Subtraction of the *Cj*TsdA nIR spectrum, on the assumption that the Heme 2 band remains relatively unchanged, suggests that the new Heme 1 His/Met CT band lies to shorter wavelength at ∼1735 nm.

Incubation with 1.5 mm ascorbate removes all significant MCD intensity at wavelengths longer than 600 nm (Fig. 7, *C* and *D*, *red*) and the spectrum is dominated by features at 425 and 552 nm that have intensities consistent with two His/Met coordinated low-spin Fe(II) hemes. Incubation with dithionite produces no further spectral changes of significance. We conclude that ascorbate reduces both hemes of *Cj*TsdA C138M and that methionine ligation is retained on reduction.

Cyclic voltammetry of *Cj*TsdA C138M at 10 mV s^−1^ (Fig. 8*C*, *black line*) corroborates the MCD in placing *E_m_* values above ≈0 V for the hemes in this protein. Peaks corresponding to oxidation and reduction are resolved between −50 and +300 mV and both have structure indicative of two overlapping contributions. Indeed, each peak is well-described by the sum (*blue circles*) of two contributions (*blue lines*) having approximately equal areas, arising from independent *n* = 1 centers with *E_m_* values of ≈+94 and ≈+174 mV. Comparison to the properties of *Cj*TsdA suggests Heme 1 has the lower and Heme 2 the higher of these *E_m_* values. Integration of the peaks confirmed *Q*_tot_^ox^ ≈ *Q*_tot_^red^ and similar values were resolved for scan rates between 5 and 100 mV s^−1^ (Fig. 6 and Fig. S3). Thus, protein film voltammetry of *Cj*TsdA C138M differs from that of the *Cj*TsdA and *Cj*TsdA N254K proteins in presenting data immediately reconciled with the presence of equal populations of electroactive Heme 1 and Heme 2.

### Spectroscopic and electrochemical characterization of CjTsdA C138H

The MCD Soret feature of oxidized *Cj*TsdA C138H (Fig. 7*E*, *black*) shows the same general characteristics as *Cj*TsdA C138M. The hemes are again both low-spin Fe(III) without thiolate ligation. Alongside the Heme 2 His/Met CT band at 1820 nm, the nIR MCD (Fig. 7*F*, *black*) contains a CT feature at 1480 nm characteristic of bis-nitrogenous coordination and indicating that His^138^ has replaced Cys^−^ as a ligand to Heme 1. Incubation with ascorbate leads to loss of the 1820 nm feature (Fig. 7*F*, *red*) consistent with reduction of Heme 2. In the absence of the sideband to this feature, the 1480 nm band has an intensity of ∼1.0 m^−1^ cm^−1^ T^−1^, consistent with one heme and ruling out any significant reduction of His/His ligated Heme 1 by ascorbate. Incubation with dithionite (Fig. 7*E*, *blue*) removes all spectral features characteristic of Fe(III) heme and leads to an increase in the peak to trough intensity of the 553 nm feature from 240 to 640 m^−1^ cm^−1^ T^−1^. Thus the contribution from His/Met Fe(II) Heme 2 is 240 m^−1^ cm^−1^ T^−1^, which is similar to the average of 220 m^−1^ cm^−1^ T^−1^ observed for the two hemes in the MCD of reduced *Cj*TsdA C138M. In contrast, the Fe(II) His/His Heme 1 in this C138H variant gives a contribution of 400 m^−1^ cm^−1^ T^−1^.

Cyclic voltammetry of *Cj*TsdA C138H at 10 mV s^−1^ (Fig. 8*D*) confirmed the presence of hemes with *E_m_* values either side of ≈0 V. The peaks at lower potential have δ ≈ 100 mV and are assigned to His/His Heme 1 with *E_m_* ≈ −100 mV. The peaks at higher potential have similar values for δ and are assigned to His/Met Heme 2 with *E_m_* ≈ +160 mV. We therefore conclude that the nature of the axial ligands is the major determinant of the Heme 1 *E_m_* value being elevated close to that of Heme 2 in *Cj*TsdA C138M and remaining in the same range (−60 to −350 mV) as for the WT proteins in *Cj*TsdA C138H.

A notable feature of the 10 mV s^−1^ voltammetry of *Cj*TsdA C138H is that the peak areas (Table S3) reveal the population of electroactive His/His ligated Heme 1 to be ∼50% of those for His/Met ligated Heme 2. However, this aspect of the voltammetry was scan rate dependent (Fig. S3 and Fig. 6). At 5 mV s^−1^ all peaks have equal area indicating equal electroactive populations of His/His ligated Heme 1 and His/Met ligated Heme 2: *Q*_hi_^ox^/*Q*_lo_^ox^ ≈ *Q*_hi_^red^/*Q*_lo_^red^ ≈ 1. At higher scan rates, the number of electrons exchanged with His/Met ligated Heme 2 was unchanged but fewer electrons were exchanged with His/His ligated Heme 1: *Q*_hi_^ox^/*Q*_lo_^ox^ ≈ *Q*_hi_^red^/*Q*_lo_^red^ < 1.

## Discussion

The complementary information afforded by MCD and PFE has provided comprehensive structural and thermodynamic descriptions of the hemes in the as purified *Av*TsdA and *Cj*TsdA enzymes. Hemes with three different axial ligand sets are present in the WT enzymes. One set, the His/Met pairing, occurs in monoheme cytochromes (32, 33) and as an electron transfer center in multiheme enzymes, including the SoxAX enzymes that perform the initial step in a thiosulfate oxidation to sulfate (14, 29). The *E_m_* value for the Fe(III) ⇌ (II) couple of Heme 2 in *Av*TsdA (+266 mV) falls within the +200 to +380 mV range observed for numerous Class I cytochromes *c* that operate as electron shuttles in photosynthetic and respiratory systems. Although *E_m_* for Heme 2 in *Cj*TsdA (+172 mV) lies below this range, there are precedents for His/Met coordination with values between +60 and +165 mV in several disparate proteins such as cyt. *bd*, cellobiose oxidase, cyt. *b*_562_, and DosH (34–38). However, compared with His/Met heme ligation, the additional ligand sets of the TsdA enzymes are significantly less common and would imply a degree of specialization.

The His/Cys^−^ coordination found for Heme 1 in the TsdA enzymes is rare and, although the purpose of such ligation is not always clear (39), some examples are suggested to function as redox sensors (40–42) and others as receptors or regulators (43–47). Reported *E_m_* values lie in the range −350 to −160 mV (40–43, 47). Considering only the Heme 1 *E_m_* values, which fall within this low range, it could be concluded that both TsdA enzymes should be biased to perform tetrathionate reduction more rapidly than thiosulfate oxidation. Such a conclusion is clearly at odds with the catalytic properties of the purified enzymes (Fig. 1) and the cellular role of *Av*TsdA (3). However, prior to the discovery of the TsdAs, His/Cys^−^ centers had also been identified in the active sites of SoxAX enzymes, where they display reversible Fe(III) ⇌ Fe(II) transitions with *E_m_* values at or below −400 mV (14, 29, 48). Although these potentials are markedly more negative than those reported here for Heme 1, it has been proposed that conjugation of thiosulfate to the cysteinate ligand, forming a cysteinyl thiosulfate and releasing two electrons, is an initiation step common to SoxAX (49) and TsdA (4, 6) (in the context of SoxAX and TsdA, −SSO_3_^−^ has been referred to as a “thiosulfonate” modification) (5, 6, 8, 50). Dissociation of the ligand, concurrent with this modification, is likely to raise the heme reduction potential, facilitating transfer of the electrons to the pair of hemes in the respective enzymes. A rise in the *E_m_* of Heme 1 such that the potentials of both hemes are comparable with that of the tetrathionate/thiosulfate couple would be consistent with the facile thiosulfate oxidation displayed by both *Cj*TsdA and *Av*TsdA with application of only small overpotentials (1). A Cys → Ala substitution at the active site of *Starkeya novella* SoxAX suggests that the magnitude of this elevation in *E_m_* could exceed 500 mV (51). The SoxAX and TsdA enzymes would then differ in the nature of the subsequent, and redox neutral, steps during which the −SSO_3_^−^ moiety of the cysteinyl thiosulfate is transferred either to the cysteinate of the SoxYZ protein in the case of SoxAX or to a second thiosulfate with TsdA The generally low heme *E_m_* values we observe for Heme 1 are likely to be a straightforward consequence of cysteinate ligation (52, 53), whereas the 200–300 mV difference between SoxAX and TsdA may reflect characteristics of the distal pockets that promote the transfer of −SSO_3_^−^ to distinct second substrates.

The reactivity of free thiolate makes it susceptible to (SO_x_) modifications (54). TsdA (and SoxAX) may have evolved a dual role for Heme 1 in that it acts not only as an electron transfer center but also as a tether for the Cys^−^, thus offering a degree of protection from these unwanted modifications prior to reaction with substrate and concomitant dissociation. Supporting the proposal that this ligand can be chemically modified is the observation of a cysteinate sulfane (persulfide) modification in structures reported for *Av*TsdA (4). It remains a possibility, for TsdA, that −SSO_3_^−^ addition occurs not to cysteine but to cysteine persulfide, as has been reported for SoxAX (50).

Furthermore, exposure to thiosulfate or tetrathionate results in the formation of high-spin heme, presumed to be Heme 1 lacking the Cys ligand, and a +112 mass increase indicative of thiosulfate conjugation (6). The structure of the as-isolated TsdA from *Marichromatium purpuratum* (8) reveals a dissociated cysteinate already bearing the −SSO_3_^−^ modification. Intriguingly, this enzyme is fused to a di-heme electron acceptor, raising the possibility that rapid electron transfer out of Hemes 1 and 2 mitigates against artifactual re-reduction of the −SSO_3_^−^ moiety to yield cysteine persulfide and sulfite.

His/Lys, the second unusual heme ligation naturally occurrent in *Av*TsdA, and engineered into *Cj*TsdA N254K, results in significantly lower Heme 2 midpoint potentials than those observed for the His/Met forms. Although a lowering of potential is consistent with replacing a thioether with an amine ligand (55) and is typical for Met to Lys substitution (56, 57), it is unlikely that the novel lysine ligation in *Av*TsdA is present simply to lower the value of *E_m_*. A potential of −129 mV is well within the range that can be achieved using His/His coordination (58, 59). Although switching Heme 2 to a lower potential may be the purpose of displacing the methionine, the selection of lysine over histidine may assist dissociation. Lability of the lysine ligand is a common characteristic of the limited number of His/Lys-coordinated hemes reported to date (60–63). However, in rapid turnover, Heme 2 would remain His/Lys coordinated in *Av*TsdA. The retention of an accessible His/Met form appears not to be critical for catalysis. In contrast, Heme 2 in *Cj*TsdA N254K remains His/Met after the first turnover and this may explain why tetrathionate reduction is not compromised in this variant.

The limiting values we have obtained for the rates of ligand exchange would imply that lysine remains bound during turnover. But the low *E_m_* value determined for the His/Lys ligand set (−171 mV) precludes reduction by the substrate thiosulfate. The possibility that lysine ligation is in some way responsible for the pronounced directionality of the *Av* enzyme can also be ruled out because the *Av*K208N variant displays the same residual tetrathionate reductase activity as the WT enzyme (4). The exact purpose of the His/Lys ligation at Heme 2 therefore remains to be determined. Before closing, we return to consider the cyclic voltammetry of *Cj*TsdA that suggests His/Cys^−^ Heme 1 has a lower electroactive population than that of His/Met Heme 2. This is surprising given that, based on the structure of *Av*TsdA, a heme edge-to-edge distance of 8 Å is predicted such that fast electron transfer is expected (64). However, there is an indication that heme-heme electron transfer may be more complex in the *Cj*TsdA enzyme from the cyclic voltammetry of *Cj*TsdA C138H. This reveals that, at increasingly high scan rate, the electroactivity of Heme 1 is tuned out, whereas that of Heme 2 is unchanged in behavior indicative of relatively slow heme-heme electron exchange. Other possibilities can be proposed to account for the voltammetry of *Cj*TsdA, for example Heme 1 exists in a number of forms having different *E_m_* values and contributing small unresolved features to the voltammetry. This situation could arise from modification(s) of the Cys^−^, perhaps relevant to catalysis, although at present this suggestion appears to be at odds with the MCD ligand assignments.

In conclusion our studies have demonstrated the ability for MCD in combination with PFE to provide chemically detailed descriptions of TsdA redox activity. Our ongoing experiments aim to exploit this approach to better understand the catalytic mechanism(s) and relative activities of the different TsdA family members. For example, to resolve the redox properties of these enzymes in the presence of their natural redox partners, HiPIP for *Av*TsdA (65) and a monoheme cytochrome *c* for *Cj*TsdA (3), and with Cys^−^ modifications that are proposed catalytic intermediates.

## Experimental procedures

### Protein preparation

WT and variant TsdAs were produced as described in Ref. 4 (for *Av*TsdA) and Ref. 5 (for *Cj* proteins). Recombinant *Cj*TsdA contains the 309 amino acids predicted from C8J_0815 without the signal peptide and with an N terminus having a Strep II-tag preceded by three amino acids and followed by four amino acids such that the predicted mass of the mature protein with two *c*-hemes is 37103 Da. Recombinant *Av*TsdA is comprised of 243 amino acids predicted from Alvin_0091 without signal peptide followed by a C-terminal extension of one additional amino acid and the Strep-tag (predicted mass of mature protein 28142 Da with two *c*-hemes). The amino acids of the *Cj* proteins are numbered from the first of the three residues preceding the Strep II-tag (5) and for *Av*TsdA from the first residue of the mature protein (4).

### Magnetic circular dichroism

MCD spectra were recorded using circular dichrographs, JASCO models J810 for the wavelength range 250–800 nm and J730 for the range 800–2000 nm. An 8 T magnetic field was generated using an Oxford Instruments Special Spectromag 1000 split coil superconducting solenoid with a 50-mm ambient temperature bore. Samples were exchanged into 50 mm HEPES, 50 mm NaCl at pH 7.0 for spectra in the range 250–800 nm and pH* 7.0 for spectra in the range 800–2000 nm (pH* being the apparent pH recorded using a glass electrode for a solution prepared in D_2_O). Sample concentrations were as indicated in the figure legends and were calculated from the absorption Soret maximum (extinction coefficients shown in Table S1). UV-visible electronic absorbance spectra of protein samples were recorded using a Hitachi model 4100 UV-visible-nIR spectrophotometer. Spectra of the oxidized enzymes are for the as-prepared samples unless traces of autoreduction were apparent from the absorbance spectra, in which case substoichiometric quantities of potassium ferricyanide were titrated into the sample until the spectrum showed the protein to be fully oxidized.

Semireduced and fully reduced samples for MCD were prepared in a N_2_ chamber (atmospheric O_2_ < 10 ppm) by adding small aliquots of concentrated solutions (∼100 mm) of sodium ascorbate and subsequently sodium dithionite until no further changes were observed in the absorption spectrum. Final concentrations of 1.5 mm, both for ascorbate and for dithionite, were sufficient for all samples with the exception of *Cj*TsdA N254K, which required 5 mm dithionite for full reduction. All potentials here, and throughout the manuscript, are quoted *versus* SHE. Although the reduction potential for the ascorbyl radical anion/ascorbate couple is *E*^o^′ = +330 mV, a low concentration of the radical is maintained by rapid disproportionation and consequently ascorbate solutions produce an effective potential in the region of +60 mV (66, 67). This was verified here by determining a value of +59 ± 12 mV for a 1.5 mm solution of sodium ascorbate, using a combination electrode placed in a solution containing a mediator mixture (2 μm each) of 3,6-diaminodurene (DAD) (*E_m_* = +276 mV); 2-((3-(3,6-dichloro-9H-carbazol-9-yl)-2-hydroxypropyl)amino)-2-(hydroxymethyl)propane-1,3-diol (DCAP) (*E_m_* = +217 mV); 2,6-dichloroindophenol sodium salt hydrate (DCPIP) (*E_m_* = +217 mV); phenazine methosulfate (PMS) (*E_m_* = +80 mV); phenazine ethosulfate (PES) (*E_m_* = +55 mV); juglone (*E_m_* = +30 mV); methylene blue (*E_m_* = +11 mV); duraquinone (*E_m_* = +5 mV); menadione (*E_m_* = −70 mV); indigo carmine (*E_m_* = −125 mV); anthraquinone-2,6-disulfonic acid disodium salt (ADQS) (*E_m_* = −185 mV); anthraquinone-2-sulfonic acid sodium salt monohydrate (AQS) (*E_m_* = −225 mV); phenosafranine (*E_m_* = −252 mV); safranine-O (*E_m_* = −280 mV); benzyl viologen (*E_m_* = −350 mV); and methyl viologen (*E_m_* = −440 mV). At pH 7.0, the concentrations of dithionite used produce potentials in the region of −500 mV (68).

### PFE of TsdA

Working electrodes (30) were comprised of inverse-opal indium-tin oxide (IO-ITO) (20 μm thickness, 0.25 cm^2^ footprint (geometrical surface area), and 750 nm pore diameter) on fluoride-doped tin oxide–coated glass. Solutions containing 50–100 μm protein (50 mm HEPES, 50 mm NaCl, pH 7) and 1.25 mm neomycin sulfate were drop cast onto ice-cold electrodes and left for 20 min before transfer to a N_2_ chamber (atmosphere < 10 ppm O_2_) where they were rinsed (50 mm HEPES, 50 mm NaCl, pH 7) to remove loosely bound protein. Experiments were performed in the N_2_ chamber employing a three-electrode cell configuration (69) containing 50 mm HEPES, 50 mm NaCl, pH 7, at 4 °C. Cyclic voltammetry was performed with PGSTAT12 and PGSTAT30 potentiostats (Metrohm Autolab) under the control of NOVA 1.11 software. Subtraction of an appropriate baseline response from the measured voltammograms (see Fig. S2) was performed using the NOVA software prior to data analysis. Protein (Faradaic) responses were fit assuming each redox-active center displays Nernstian behavior (31) for which the oxidative (reductive) peak has the form in Equation 2:

(Eq. 2) $$\left|i\left(E\right)\right|=\frac{n^{2}F^{2}}{RT}\frac{\nu \Gamma_{\text{O}}^{*}\exp\left(nF\left(E-E_{m}\right)/RT\right)}{{\left(1+\exp\left(nF\left(E-E_{m}\right)/R\right)\right)}^{2}}$$

where *i(E*) is current as a function of electrode potential *E*; ν is the scan rate; Γ_o_^*^ is the population of adsorbed redox center; *R*, *F*, and *T* have their usual meanings; and the number of electrons transferred in the half-reaction (*n*) is 1. For TsdA hemes, the *E_m_* values reported herein are averages obtained from the corresponding oxidative and reductive peaks.

## Author contributions

L. P. J., J. N. B., and M. R. C. data curation; L. P. J., J. M. B., J. N. B., and M. R. C. formal analysis; L. P. J., J. M. B., and M. R. C. investigation; L. P. J., J. N. B., and M. R. C. visualization; L. P. J., J. M. K., K. P. S., E. R., C. D., J. M. B., J. N. B., and M. R. C. writing-review and editing; J. M. K., S. v. H., K. P. S., and E. R. resources; E. R., C. D., J. M. B., J. N. B., and M. R. C. supervision; E. R., C. D., J. N. B., and M. R. C. funding acquisition; C. D., J. M. B., J. N. B., and M. R. C. conceptualization; J. M. B., J. N. B., and M. R. C. writing-original draft; J. N. B. and M. R. C. validation; J. N. B. and M. R. C. project administration.

## Supplementary Material

Supporting Information

## Footnotes

6 The abbreviations used are:

TsdA: thiosulfate dehydrogenase
*Av*: *Allochromatium vinosum*
*Cj*: *Campylobacter jejuni*
CT: charge transfer
MCD: magnetic CD
PFE: protein film electrochemistry
pH*: the apparent pH recorded using a glass electrode for a solution prepared in D_2_O
SHE: standard hydrogen electrode
nIR: near infrared.

## References

[1] Kurth J. M., Dahl C., and Butt J. N. (2015) Catalytic protein film electrochemistry provides a direct measure of the tetrathionate/thiosulfate reduction potential. J. Am. Chem. Soc. 137, 13232–13235 10.1021/jacs.5b08291 26437022

[2] Denkmann K., Grein F., Zigann R., Siemen A., Bergmann J., van Helmont S., Nicolai A., Pereira I. A. C., and Dahl C. (2012) Thiosulfate dehydrogenase: A widespread unusual acidophilic *c*-type cytochrome. Environ. Microbiol. 14, 2673–2688 10.1111/j.1462-2920.2012.02820.x 22779704

[3] Liu Y. W., Denkmann K., Kosciow K., Dahl C., and Kelly D. J. (2013) Tetrathionate stimulated growth of *Campylobacter jejuni* identifies a new type of bi-functional tetrathionate reductase (TsdA) that is widely distributed in bacteria. Mol. Microbiol. 88, 173–188 10.1111/mmi.12176 23421726

[4] Brito J. A., Denkmann K., Pereira I. A. C., Archer M., and Dahl C. (2015) Thiosulfate dehydrogenase (TsdA) from *Allochromatium vinosum.* Structural and functional insights into thiosulfate oxidation. J. Biol. Chem. 290, 9222–9238 10.1074/jbc.M114.623397 25673691 PMC4423707

[5] Kurth J. M., Butt J. N., Kelly D. J., and Dahl C. (2016) Influence of haem environment on the catalytic properties of the tetrathionate reductase TsdA from *Campylobacter jejuni*. Biosci. Rep. 36, e00422 10.1042/BSR20160457 27789780 PMC5146829

[6] Grabarczyk D. B., Chappell P. E., Eisel B., Johnson S., Lea S. M., and Berks B. C. (2015) Mechanism of thiosulfate oxidation in the SoxA family of cysteine-ligated cytochromes. J. Biol. Chem. 290, 9209–9221 10.1074/jbc.M114.618025 25673696 PMC4423706

[7] Kurth J. M., Schuster A., Seel W., Herresthal S., Simon J., and Dahl C. (2017) TsdC, a unique lipoprotein from *Wolinella succinogenes* that enhances tetrathionate reductase activity of TsdA. FEMS Microbiol. Lett. 364, fnx003 10.1093/femsle/fnx003 28062520

[8] Kurth J. M., Brito J. A., Reuter J., Flegler A., Koch T., Franke T., Klein E. M., Rowe S. F., Butt J. N., Denkmann K., Pereira I. A. C., Archer M., and Dahl C. (2016) Electron accepting units of the diheme cytochrome *c* TsdA, a bifunctional thiosulfate dehydrogenase/tetrathionate reductase. J. Biol. Chem. 291, 24804–24818 10.1074/jbc.M116.753863 27694441 PMC5122753

[9] Gadsby P. M. A., and Thomson A. J. (1990) Assignment of the axial ligands of ferric ion in low-spin hemoproteins by near-infrared magnetic circular dichroism and electron paramagnetic resonance spectroscopy. J. Am. Chem. Soc. 112, 5003–5011 10.1021/ja00169a002

[10] Cheesman M. R., Greenwood C., and Thomson A. J. (1991) Magnetic circular dichroism of hemoproteins. in Advances in Inorganic Chemistry (Sykes A. G., ed) Vol. 36, pp. 201–255, Academic Press, San Diego, CA 10.1016/S0898-8838(08)60040-9

[11] McKnight J., Cheesman M. R., Thomson A. J., Miles J. S., and Munro A. W. (1993) Identification of charge-transfer transitions in the optical spectrum of low-spin ferric cytochrome P-450 *Bacillus megaterium*. Eur. J. Biochem. 213, 683–687 10.1111/j.1432-1033.1993.tb17808.x 8386633

[12] Dhawan I. K., Shelver D., Thorsteinsson M. V., Roberts G. P., and Johnson M. K. (1999) Probing the heme axial ligation in the CO-sensing CooA protein with magnetic circular dichroism spectroscopy. Biochemistry 38, 12805–12813 10.1021/bi991303c 10504250

[13] Cheesman M. R., Little P. J., and Berks B. C. (2001) Novel heme ligation in a *c*-type cytochrome involved in thiosulfate oxidation: EPR and MCD of SoxAX from *Rhodovulum sulfidophilum*. Biochemistry 40, 10562–10569 10.1021/bi0100081 11523998

[14] Kappler U., Bernhardt P. V., Kilmartin J., Riley M. J., Teschner J., McKenzie K. J., and Hanson G. R. (2008) SoxAX cytochromes, a new type of heme copper protein involved in bacterial energy generation from sulfur compounds. J. Biol. Chem. 283, 22206–22214 10.1074/jbc.M800315200 18552405

[15] Kilmartin J. R., Maher M. J., Krusong K., Noble C. J., Hanson G. R., Bernhardt P. V., Riley M. J., and Kappler U. (2011) Insights into structure and function of the active site of SoxAX cytochromes. J. Biol. Chem. 286, 24872–24881 10.1074/jbc.M110.212183 21592966 PMC3137062

[16] Dawson J. H., Andersson L. A., and Sono M. (1982) Spectroscopic investigations of ferric cytochrome P-450-CAM ligand complexes. Identification of the ligand *trans* to cysteinate in the native enzyme. J. Biol. Chem. 257, 3606–3617 6277939

[17] Berka V., Palmer G., Chen P. F., and Tsai A. L. (1998) Effects of various imidazole ligands on heme conformation in endothelial nitric oxide synthase. Biochemistry 37, 6136–6144 10.1021/bi980133l 9558353

[18] Shimizu T., Nozawa T., Hatano M., Imai Y., and Sato R. (1975) Magnetic circular dichroism studies of hepatic microsomal cytochrome P-450. Biochemistry 14, 4172–4178 10.1021/bi00690a004 1182096

[19] Vickery L., Salmon A., and Sauer K. (1975) Magnetic circular dichroism studies on microsomal aryl hydrocarbon hydroxylase: Comparison with cytochrome *b*_5_ and cytochrome P-450_cam_. Biochim. Biophys. Acta 386, 87–98 10.1016/0005-2795(75)90249-4 164936

[20] Dawson J. H., Trudell J. R., Linder R. E., Barth G., Bunnenberg E., and Djerassi C. (1978) Magnetic circular dichroism of purified forms of rabbit liver cytochromes P-450 and P-420. Biochemistry 17, 33–42 10.1021/bi00594a006 618545

[21] Dawson J. H., Sono M., and Hager L. P. (1983) The active sites of chloroperoxidase and cytochrome P-450-CAM: Comparative spectroscopic and ligand binding properties. Inorg. Chim. Acta 79, 184–186 10.1016/S0020-1693(00)95217-4

[22] Shimizu T., Iizuka T., Mitani F., Ishimura Y., Nozawa T., and Hatano M. (1981) Magnetic and natural circular dichroism spectra of cytochromes *P*-450_11β and_ *P*-450_SCC_ purified from bovine adrenal cortex. Biochim. Biophys. Acta 669, 46–59 10.1016/0005-2795(81)90222-1 7295771

[23] Svastits E. W., Alberta J. A., Kim I. C., and Dawson J. H. (1989) Magnetic circular dichroism studies of the active site structure of hemoprotein H-450: Comparison to cytochrome P-450 and sensitivity to pH effects. Biochem. Biophys. Res. Commun. 165, 1170–1176 10.1016/0006-291X(89)92725-3 2610685

[24] Sigman J. A., Pond A. E., Dawson J. H., and Lu Y. (1999) Engineering cytochrome *c* peroxidase into cytochrome P450: A proximal effect on heme-thiolate ligation. Biochemistry 38, 11122–11129 10.1021/bi990815o 10460168

[25] Rux J. J., and Dawson J. H. (1991) Magnetic circular dichroism spectroscopy as a probe of axial heme ligand replacement in semisynthetic mutants of cytochrome *c*. FEBS Lett. 290, 49–51 10.1016/0014-5793(91)81222-/T 1655536

[26] Simpkin D., Palmer G., Devlin F. J., McKenna M. C., Jensen G. M., and Stephens P. J. (1989) The axial ligands of heme in cytochromes: A near-infrared magnetic circular dichroism study of yeast cytochromes *c*, *c*_1_, and *b* and spinach cytochrome *f*. Biochemistry 28, 8033–8039 10.1021/bi00446a010 2557894

[27] Rigby S. E. J., Moore G. R., Gray J. C., Gadsby P. M. A., George S. J., and Thomson A. J. (1988) NMR, EPR and magnetic CD studies of cytochrome *f.* Identity of the haem axial ligands. Biochem. J. 256, 571–577 10.1042/bj2560571 3223931 PMC1135448

[28] Gadsby P. M. A., Peterson J., Foote N., Greenwood C., and Thomson A. J. (1987) Identification of the ligand-exchange process in the alkaline transition of horse heart cytochrome *c*. Biochem. J. 246, 43–54 10.1042/bj2460043 2823795 PMC1148238

[29] Bradley J. M., Marritt S. J., Kihlken M. A., Haynes K., Hemmings A. M., Berks B. C., Cheesman M. R., and Butt J. N. (2012) Redox and chemical activities of the hemes in the sulfur oxidation pathway enzyme SoxAX. J. Biol. Chem. 287, 40350–40359 10.1074/jbc.M112.396192 23060437 PMC3504750

[30] Mersch D., Lee C. Y., Zhang J. Z., Brinkert K., Fontecilla-Camps J. C., Rutherford A. W., and Reisner E. (2015) Wiring of photosystem II to hydrogenase for photoelectrochemical water splitting. J. Am. Chem. Soc. 137, 8541–8549 10.1021/jacs.5b03737 26046591

[31] Bard A. J., and Faulkner L. R. (2000) Electrochemical Methods: Fundamentals and Applications, 2nd ed., John Wiley & Sons, Somerset, NJ

[32] Battistuzzi G., Borsari M., and Sola M. (2001) Medium and temperature effects on the redox chemistry of cytochrome *c*. Eur. J. Inorg. Chem. 2989–3004 10.1002/1099-0682(200112)2001:12<2989::AID-EJIC2989>3.0.CO;2-E

[33] Battistuzzi G., Borsari M., Cowan J. A., Ranieri A., and Sola M. (2002) Control of cytochrome *c* redox potential: Axial ligation and protein environment effects. J. Am. Chem. Soc. 124, 5315–5324 10.1021/ja017479v 11996572

[34] Springs S. L., Bass S. E., Bowman G., Nodelman I., Schutt C. E., and McLendon G. L. (2002) A multigeneration analysis of cytochrome *b*_562_ redox variants: Evolutionary strategies for modulating redox potential revealed using a library approach. Biochemistry 41, 4321–4328 10.1021/bi012066s 11914078

[35] Barker P. D., Nerou E. P., Cheesman M. R., Thomson A. J., de Oliveira P., and Hill H. A. O. (1996) Bis-methionine ligation to heme iron in mutants of cytochrome *b*_562_. 1. Spectroscopic and electrochemical characterization of the electronic properties. Biochemistry 35, 13618–13626 10.1021/bi961127x 8885841

[36] Koland J. G., Miller M. J., and Gennis R. B. (1984) Potentiometric analysis of the purified cytochrome *d* terminal oxidase complex from *Escherichia coli*. Biochemistry 23, 1051–1056 10.1021/bi00301a003

[37] Ludwig R., Ortiz R., Schulz C., Harreither W., Sygmund C., and Gorton L. (2013) Cellobiose dehydrogenase modified electrodes: Advances by materials science and biochemical engineering. Anal. Bioanal. Chem. 405, 3637–3658 10.1007/s00216-012-6627-x 23329127 PMC3608873

[38] Sasakura Y., Hirata S., Sugiyama S., Suzuki S., Taguchi S., Watanabe M., Matsui T., Sagami I., and Shimizu T. (2002) Characterization of a direct oxygen sensor heme protein from *Escherichia coli.* Effects of the heme redox states and mutations at the heme-binding site on catalysis and structure. J. Biol. Chem. 277, 23821–23827 10.1074/jbc.M202738200 11970957

[39] Saha R., Bose M., Sen Santara S., Roy J., and Adak S. (2013) Identification of proximal and distal axial ligands in *Leishmania major* pseudoperoxidase. Biochemistry 52, 8878–8887 10.1021/bi401343t 24261670

[40] Singh S., Madzelan P., Stasser J., Weeks C. L., Becker D., Spiro T. G., Penner-Hahn J., and Banerjee R. (2009) Modulation of the heme electronic structure and cystathionine β-synthase activity by second coordination sphere ligands: The role of heme ligand switching in redox regulation. J. Inorg. Biochem. 103, 689–697 10.1016/j.jinorgbio.2009.01.009 19232736 PMC2772092

[41] Motomura T., Suga M., Hienerwadel R., Nakagawa A., Lai T. L., Nitschke W., Kuma T., Sugiura M., Boussac A., and Shen J. R. (2017) Crystal structure and redox properties of a novel cyanobacterial heme protein with a His/Cys heme axial ligation and a Per-Arnt-Sim (PAS)-like domain. J. Biol. Chem. 292, 9599–9612 10.1074/jbc.M116.746263 28428249 PMC5465485

[42] Alric J., Tsukatani Y., Yoshida M., Matsuura K., Shimada K., Hienerwadel R., Schoepp-Cothenet B., Nitschke W., Nagashima K. V. P., and Vermeglio A. (2004) Structural and functional characterization of the unusual triheme cytochrome bound to the reaction center of *Rhodovulum sulfidophilum*. J. Biol. Chem. 279, 26090–26097 10.1074/jbc.M400361200 15069076

[43] Nakajima H., Honma Y., Tawara T., Kato T., Park S. Y., Miyatake H., Shiro Y., and Aono S. (2001) Redox properties and coordination structure of the heme in the CO-sensing transcriptional activator CooA. J. Biol. Chem. 276, 7055–7061 10.1074/jbc.M003972200 11096066

[44] Marvin K. A., Reinking J. L., Lee A. J., Pardee K., Krause H. M., and Burstyn J. N. (2009) Nuclear receptors *Homo sapiens* Rev-erbβ and *Drosophila melanogaster* E75 are thiolate-ligated heme proteins which undergo redox-mediated ligand switching and bind CO and NO. Biochemistry 48, 7056–7071 10.1021/bi900697c 19405475 PMC2849663

[45] de Rosny E., de Groot A., Jullian-Binard C., Gaillard J., Borel F., Pebay-Peyroula E., Fontecilla-Camps J. C., and Jouve H. M. (2006) *Drosophila* nuclear receptor E75 is a thiolate hemoprotein. Biochemistry 45, 9727–9734 10.1021/bi060537a 16893174

[46] Joshi M., Kulkarni A., and Pal J. K. (2013) Small molecule modulators of eukaryotic initiation factor 2α kinases, the key regulators of protein synthesis. Biochimie 95, 1980–1990 10.1016/j.biochi.2013.07.030 23939221

[47] Grein F., Venceslau S. S., Schneider L., Hildebrandt P., Todorovic S., Pereira I. A. C., and Dahl C. (2010) DsrJ, an essential part of the DsrMKJOP transmembrane complex in the purple sulfur bacterium *Allochromatium vinosum*, is an unusual triheme cytochrome *c*. Biochemistry 49, 8290–8299 10.1021/bi1007673 20726534

[48] Reijerse E. J., Sommerhalter M., Hellwig P., Quentmeier A., Rother D., Laurich C., Bothe E., Lubitz W., and Friedrich C. G. (2007) The unusal redox centers of SoxXA, a novel *c*-type heme-enzyme essential for chemotrophic sulfur-oxidation of *Paracoccus pantotrophus*. Biochemistry 46, 7804–7810 10.1021/bi7003526 17547421

[49] Bamford V. A., Bruno S., Rasmussen T., Appia-Ayme C., Cheesman M. R., Berks B. C., and Hemmings A. M. (2002) Structural basis for the oxidation of thiosulfate by a sulfur cycle enzyme. EMBO J. 21, 5599–5610 10.1093/emboj/cdf566 12411478 PMC131063

[50] Grabarczyk D. B., and Berks B. C. (2017) Intermediates in the Sox sulfur oxidation pathway are bound to a sulfane conjugate of the carrier protein SoxYZ. PLoS One 12, e0173395 10.1371/journal.pone.0173395 28257465 PMC5336275

[51] Kilmartin J. R., Bernhardt P. V., Dhouib R., Hanson G. R., Riley M. J., and Kappler U. (2016) Effects of mutations in active site heme ligands on the spectroscopic and catalytic properties of SoxAX cytochromes. J. Inorg. Biochem. 162, 309–318 10.1016/j.jinorgbio.2016.04.015 27112898

[52] Mowat C. G., Miles C. S., Munro A. W., Cheesman M. R., Quaroni L. G., Reid G. A., and Chapman S. K. (2000) Changing the heme ligation in flavocytochrome *b*_2_: Substitution of histidine-66 by cysteine. J. Biol. Inorg. Chem. 5, 584–592 10.1007/s007750000141 11085649

[53] Raphael A. L., and Gray H. B. (1991) Semisynthesis of axial-ligand (position 80) mutants of cytochrome *c*. J. Am. Chem. Soc. 113, 1038–1040 10.1021/ja00003a045

[54] Go Y. M., Chandler J. D., and Jones D. P. (2015) The cysteine proteome. Free Radical Biol. Med. 84, 227–245 10.1016/j.freeradbiomed.2015.03.022 25843657 PMC4457640

[55] Lever A. B. P. (1990) Electrochemical parameterization of metal complex redox potentials, using the ruthenium(III)/ruthenium(II) couple to generate a ligand electrochemical series. Inorg. Chem. 29, 1271–1285 10.1021/ic00331a030

[56] Ubbink M., Campos A. P., Teixeira M., Hunt N. I., Hill H. A. O., and Canters G. W. (1994) Characterization of mutant Met100Lys of cytochrome *c-550* from *Thiobacillus versutus* with lysine-histidine heme ligation. Biochemistry 33, 10051–10059 10.1021/bi00199a032 8060974

[57] Bradley J. M., Silkstone G., Wilson M. T., Cheesman M. R., and Butt J. N. (2011) Probing a complex of cytochrome *c* and cardiolipin by magnetic circular dichroism spectroscopy: Implications for the initial events in apoptosis. J. Am. Chem. Soc. 133, 19676–19679 10.1021/ja209144h 22081937

[58] Zheng Z., and Gunner M. R. (2009) Analysis of the electrochemistry of hemes with *E*_m_s spanning 800 mV. Proteins 75, 719–734 10.1002/prot.22282 19003997 PMC2727069

[59] Reedy C. J., Elvekrog M. M., and Gibney B. R. (2008) Development of a heme protein structure-electrochemical function database. Nucleic Acids Res. 36, D307–D313 10.1093/nar/gkm814 17933771 PMC2238922

[60] Preimesberger M. R., Majumdar A., and Lecomte J. T. J. (2017) Dynamics of lysine as a heme axial ligand: NMR analysis of the *Chlamydomonas reinhardtii* hemoglobin THB1. Biochemistry 56, 551–569 10.1021/acs.biochem.6b00926 28032976

[61] Nye D. B., Preimesberger M. R., Majumdar A., and Lecomte J. T. J. (2018) Histidine-lysine axial ligand switching in a hemoglobin: A role for heme propionates. Biochemistry 57, 631–644 10.1021/acs.biochem.7b01155 29271191 PMC6214620

[62] Teh A. H., Saito J. A., Najimudin N., and Alam M. (2015) Open and Lys-His hexacoordinated closed structures of a globin with swapped proximal and distal sites. Sci. Rep. 5, 11407 10.1038/srep11407 26094577 PMC4476040

[63] Ilcu L., Rother W., Birke J., Brausemann A., Einsle O., and Jendrossek D. (2017) Structural and functional analysis of latex clearing protein (Lcp) provides insight into the enzymatic cleavage of rubber. Sci. Rep. 7, 6179 10.1038/s41598-017-05268-2 28733658 PMC5522427

[64] Page C. C., Moser C. C., Chen X. X., and Dutton P. L. (1999) Natural engineering principles of electron tunnelling in biological oxidation-reduction. Nature 402, 47–52 10.1038/46972 10573417

[65] Fukumori Y., and Yamanaka T. (1979) High-potential non-heme iron protein (HiPIP)-linked, thiosulfate-oxidising enzyme derived from *Chromatium vinosum*. Curr. Microbiol. 3, 117–120 10.1007/BF02602443

[66] Njus D., and Kelley P. M. (1993) The secretory vesicle ascorbate regenerating system-a chain of concerted H^+^/e^−^ transfer reactions. Biochim. Biophys. Acta 1144, 235–248 10.1016/0005-2728(93)90108-R 8399278

[67] Coassin M., Tomasi A., Vannini V., and Ursini F. (1991) Enzymatic recycling of oxidised ascorbate in pig heart: one-electron *vs.* 2-electron pathway. Arch. Biochem. Biophys. 290, 458–462 10.1016/0003-9861(91)90566-2 1929413

[68] Mayhew S. G. (1978) Redox potential of dithionite and SO_2_^−^ from equilibrium reactions with flavodoxins, methyl viologen and hydrogen plus hydrogenase. Eur. J. Biochem. 85, 535–547 10.1111/j.1432-1033.1978.tb12269.x 648533

[69] Marritt S. J., Kemp G. L., Xiaoe L., Durrant J. R., Cheesman M. R., and Butt J. N. (2008) Spectroelectrochemical characterization of a pentaheme cytochrome in solution and as electrocatalytically active films on nanocrystalline metal-oxide electrodes. J. Am. Chem. Soc. 130, 8588–8589 10.1021/ja802641a 18549208

