# Supplementary material for: Heme ligation and redox chemistry in two bacterial thiosulfate dehydrogenase (TsdA) enzymes
Source: J Biol Chem. 2019 Aug 29;294(47):18002–14. doi: 10.1074/jbc.RA119.010084 (PMC6879331; doi:10.1074/jbc.RA119.010084)
Supplement: Supporting Information [file supp_294_47_18002__index.html]

Heme ligation and redox chemistry in two bacterial thiosulfate dehydrogenase (TsdA) enzymes — Heme ligation & redox chemistry in TsdA — Heme ligation and redox chemistry in two bacterial thiosulfate dehydrogenase (TsdA) enzymes — Heme ligation and redox chemistry in TsdA — Supporting Information 

# Heme ligation and redox chemistry in two bacterial thiosulfate dehydrogenase (TsdA) enzymes

## Supporting Information

- Supporting Information (to be published online) - SI
