# Supplementary material for: Heme ligation and redox chemistry in two bacterial thiosulfate dehydrogenase (TsdA) enzymes
Source: J Biol Chem. 2019 Aug 29;294(47):18002–14. doi: 10.1074/jbc.RA119.010084 (PMC6879331; doi:10.1074/jbc.RA119.010084)
Supplement: Supporting Information [file supp_RA119.010084_153928_1_supp_384534_pwsbn7.pdf]

SUPPORTING INFORMATION

Parachlamydia acanthamoebae	--	0
Campylobacter jejuni	--------------------------------LDPNL-----------------------	5
Wolinella succinogenes	--------------------------------FERWT-----------------------	5
Marichromatium purpuratum	PACVRCHGPGGVGAGAVFPPLAGQPYSYLLAQLQAWGTGRRHGEPMALMGAVAGRLDADE	180
Thiomonas intermedia	--	0
Sideroxydans lithotrophicus	--	0
Pseudomonas stutzeri	--	0
Allochromatium vinosum	--	0
Psychrobacter arcticus	---SDNTE	5

Parachlamydia acanthamoebae	----------------------------------SSN-----------------------	3
Campylobacter jejuni	--------EK----------------------TKSATGIDLPTAKWNLPKALNED-----	30
Wolinella succinogenes	--------LE----------------------ARGKSGYDAPKLEWVEGKTINEIKNPTI	35
Marichromatium purpuratum	QRALAAYFATRPLAVADPADDLPDPSPPPATASVSTPAMTVAGANAVVPEHLGAV-----	235
Thiomonas intermedia	--ADAPMAP-PKSEI-----	12
Sideroxydans lithotrophicus	---ASET-----	4
Pseudomonas stutzeri	--AEIKMDDQSQLTQ-----	13
Allochromatium vinosum	--	0
Psychrobacter arcticus	TKAVERVEEAAALA---RVKDLEA------RAEALKS--NMPAANDMTA---TAG-----	46

Parachlamydia acanthamoebae	--GMSSSNDTQEWRTTLLDPEMAPENIKPLVMQGFRILFDTKK--------HAPQYAGDE	53
Campylobacter jejuni	-------------GTIDETKMPKNSEYSKMVILGNKILNETSKYVGPQAKDPKKRFAGNN	77
Wolinella succinogenes	QYALPKFEGDVFWDTINAKPVVPDSIYGEFVQYGYELFVNTQAVIGPEVADPNMRYSGNN	95
Marichromatium purpuratum	--PAGRAEAAS-RFTPPSRDALPEGPLGEMVRLGARLFRHTNTD------PRSAPHVGND	286
Thiomonas intermedia	--NA-AVGTGA-KFTPPPESAIPDDDFGKMVKLGRDIMLDTPK--------YAKDYVGNT	60
Sideroxydans lithotrophicus	--STPTAAKAL-VFTPPNDEEIPNNEFGKIVRQGKNIFEDTQH--------YAKQYVGNS	53
Pseudomonas stutzeri	--KAGKGAGES-YFQPPQEKDLPANAYGELVQQGRAIFVDTQK--------YAAEYVGNG	62
Allochromatium vinosum	-----EEPPTV-ALTVPAAALLPDGALGESIVRGRRYLSDTPA--------QLPDFVGNG	46
Psychrobacter arcticus	--GTDTASGKP-TIKMPDESTIPDDEFGAAVRRGLQISNHTYK--------ELPNNVGNQ	95

Parachlamydia acanthamoebae	IS**C**TN**CH**FNCGNTFGGENNGISLVGVTKKYPRAILD-NPHYTLEERINA**C**FTKSLNG---	109
Campylobacter jejuni	LS**C**SS**CH**ANGG----SVQNQSGFVGIWARFPQYNARGDKVITLADRING**C**FERSMNG---	130
Wolinella succinogenes	LS**C**NS**CH**LGAG----TAKYAAPLVDNHANFPQYRNRENSLGTMAARVNG**C**MQRSMNG---	148
Marichromatium purpuratum	QT**C**AG**CH**LDNG----RRADASPMWAAWVAYPAYRGKNQRVDTMAERIQG**C**FRYSMNAQDS	342
Thiomonas intermedia	LS**C**VN**CH**TDAG----RMAGSAPLWAAYVSYPAYRGKNKKVNTFEERLQG**C**FKFSQNG---	113
Sideroxydans lithotrophicus	LN**C**VN**CH**LASG----RKENSSPLWAAYVRYPAYRAKNNKVNTYEERIQG**C**FKYSLNG---	106
Pseudomonas stutzeri	MN**C**TN**CH**LEQG----RKANSAPLWGAYPMYPAYRKKNDKVNSYAERVQG**C**FQFSMNG---	115
Allochromatium vinosum	LA**C**RH**CH**PGRDGEVGTEANAAPFVGVVGRFPQYSARHGRLITLEQRIGD**C**FERSLNG---	103
Psychrobacter arcticus	LN**C**TS**CH**LGNG----SEAYAAPWNNTPSVYPNYSKRTGRINTIQERING**C**FERSLNG---	148

Parachlamydia acanthamoebae	---KPVPLKSKEMKAMIAYLEWISKGVSNQA---PWLGLKKLRS----HAIPNAEKGNAL	159
Campylobacter jejuni	---KRMPSDTPEMKAMLTYMQWLSQGVPVGA-KIEGQGLKKIDF---ISRAADPKKGKAI	183
Wolinella succinogenes	---YPLPAEGKEMKAFLAYIHWLGQGIPVGA-KIEGRSLKTVDRKMVQQNAADVKNGAEV	204
Marichromatium purpuratum	VSGQVPETNGLVLDALQSYIFWLATGAPTGDTAMSGRGYPRLQP---PAEGFDRTRGAAL	399
Thiomonas intermedia	---KAPPLGSKTLVALESYSYWLSKGLPVDE-KVAGRGYPNLPE---PQQAPDYVRGQKV	166
Sideroxydans lithotrophicus	---KAPAVDSPEMVALVTYSYWLATGAPVGA-KLKGAGYPEVPK---PGLIPDANRGKTV	159
Pseudomonas stutzeri	---TPPAADSHVINALTAYSYWLSTGAPTGQ-ELPGRAYPEVPQ---PQGGFDIAKGKQI	168
Allochromatium vinosum	---RALALDHPALIDMLAYMSWLSQGVPVGA-VVAGHGIPTLTL---E-REPDGVHGEAL	155
Psychrobacter arcticus	---KALDLNSDDMNAMVSYMSWLSQDMPFGV-SPEGSGFVKVDK---T-LEPNTDN**G**KKL	200

Parachlamydia acanthamoebae	YQQH**C**AA**CH**GKDGEGQLRPD------NLKYPPLWGN-HSFNQAAGMNDLPTLAAFIYDN**M**	212
Campylobacter jejuni	YMDK**C**AV**CH**QENGLGLKNEDSA--GAYYLYPPLWGD-DSYNTGAGMYRLIKAASYIKEN**M**	240
Wolinella succinogenes	YARD**C**AS**CH**GAEGEGLRRESKDGKPAGYEFPPLWGSDDTYNTGAGMYRTLKAADFIKST**M**	264
Marichromatium purpuratum	YAEH**C**AL**CH**GAEGEGLL-VD-----GEVVFPPLWGP-RSYNWGAGMHRVDTAAAFIAAN**M**	452
Thiomonas intermedia	YEAK**C**IL**CH**AANGEGQY-VN-----GETVFPPLWGP-KSFNWGAGMGSYKNAAKFIYAN**M**	219
Sideroxydans lithotrophicus	FVEN**C**QV**CH**GSNGEGKK-VD-----GKYIFPPLWGS-ESFNWGAGMHRINTAAAFIKAN**M**	212
Pseudomonas stutzeri	YAEQ**C**AV**CH**GDDGQGQK-AG-----GGYVFPPLWGK-DSFNWGAGMHRINTAAAFIKES**M**	221
Allochromatium vinosum	YQAR**C**LA**CH**GADGSGTLDAD-----GRYLFPPLWGP-RSFNTGAGMNRQATAAGFIKH**KM**	209
Psychrobacter arcticus	FAEK**C**SV**CH**GATGEGQYNDD-----GTYVYPAIAGD-KSFNDGAGMARTYTAASFIKG**KM**	254

Parachlamydia acanthamoebae	PYQEP-RLTVEEVLDIAAYITSQ--PRPIEIRTP--------------------------	243
Campylobacter jejuni	PQGAP-DLSLEDAYDVAAYMNSQ--ARPIKANRDKDFPDRKVK---PLDMD---VGPYDD	291
Wolinella succinogenes	PKGAP-TLSDKDAYDVAAFINDYSHYRTVKLNRQNDFVDPKVR---VPDHDQ--PGPYGP	318
Marichromatium purpuratum	PLLDTVRLTPQEAWDVAAYINAH--ERPQDPRF----DG-SVERTAARFHASP-FDLYGE	504
Thiomonas intermedia	PYGMSYSLSPQEAWDVAYFMDAQ--ERPQDPRW----QG-SVAATRAKFHDSK-FSLYGT	271
Sideroxydans lithotrophicus	PLSKGGTLTDQEAWDVATFVMSH--ERPQDPRF----KG-NVAQTKKEYHDE--NCRYGE	263
Pseudomonas stutzeri	PLGKGGSLSDADAWHVAAYMNSH--ERPQDPRL----IEGSVEKTRLKYHANDGVNLYGQ	275
Allochromatium vinosum	PLGADDSLSDEEAWDVAGFVLTH--PRPLFQEPTGD------------------------	243
Psychrobacter arcticus	PFGQGGSLSDQEAVDIASYFTHL--PRPIKANKDKDWPNGDAPKDVRR------------	300

Parachlamydia acanthamoebae	----------------------------------	243
Campylobacter jejuni	S---------FSTTQHRYGPYTNMIKK-------	309
Wolinella succinogenes	EGSYIFPDEGKTQMDYKVGPYKGIIKQKPAAK--	350
Marichromatium purpuratum	P---------------LGVDGAVLGQGVAKD--	520
Thiomonas intermedia	K------------------VNGKLLGDIGAPKPR	287
Sideroxydans lithotrophicus	T------------------VNGKVLGGKHQTAAK	279
Pseudomonas stutzeri	Q------------------VDGALLGQGVK----	287
Allochromatium vinosum	----------------------------------	243
Psychrobacter arcticus	----------------------------------	300

Figure S1. Sequence alignment for TsdAs from *Parachlamydia acanthamoebae*, *Campylobacter jejuni*, *Wolinella succinogenes*, *Allochromatium vinosum*, *Marichromatium purpuratum*, *Thiomonas intermedia*, *Sideroxydans lithotrophicus* and *Pseudomonas stutzeri*. Heme binding motifs including proximal His ligand are indicated in blue with the distal Cys ligand to Heme 1 in red and M(K) ligand to Heme 2 in brown.

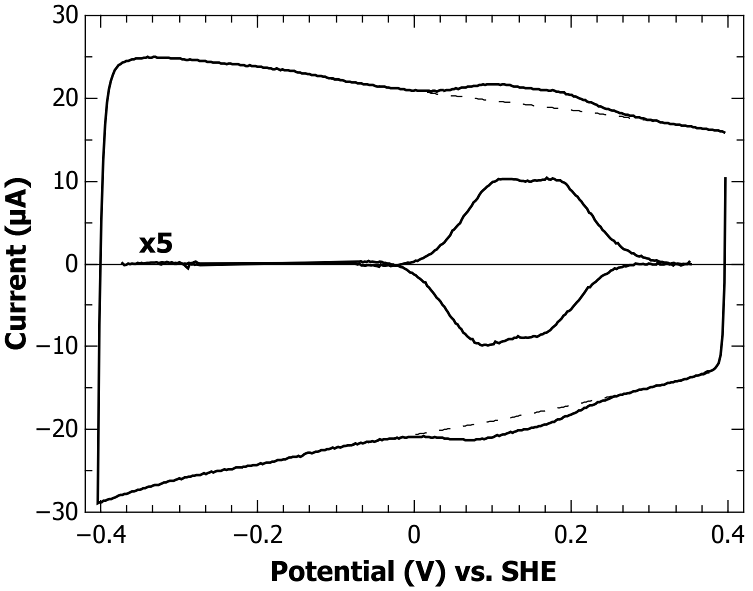

Figure S2. Representative cyclic voltammetry of a TsdA protein adsorbed on IO-ITO. Voltammogram (continuous line) is for *Cj*TsdA C138M in 50 mM HEPES, 50 mM NaCl, pH 7, 4 °C measured at 10 mV s^{-1}. Inset: the protein (Faradaic) response obtained by subtraction of a baseline (dashed line) from the measured current.

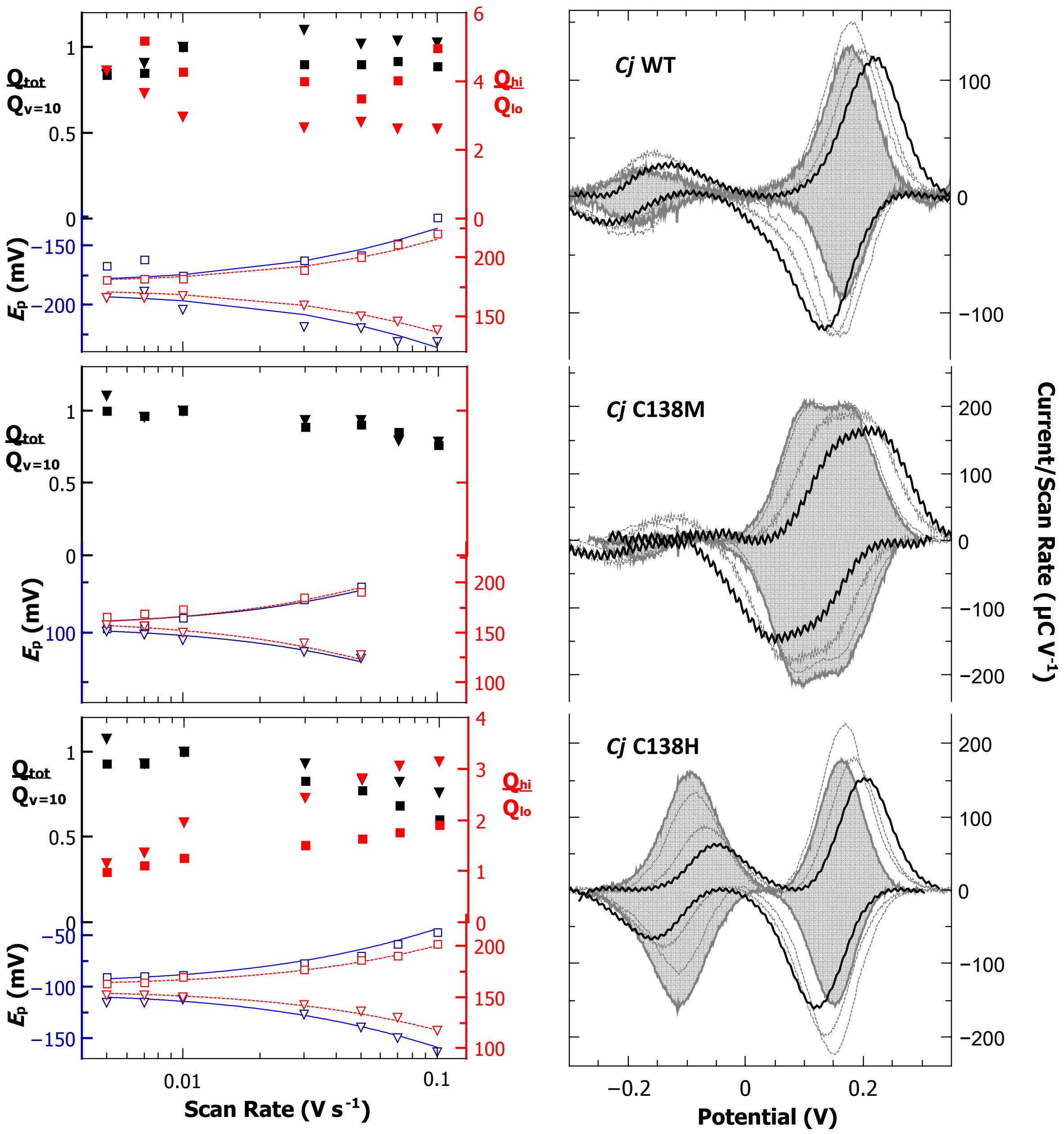

Figure S3. Scan-rate dependence of the cyclic voltammetry for *Cj*TsdA and Heme 1 variants adsorbed on IO-ITO electrodes.

(Right) Representative baseline subtracted cyclic voltammograms for the indicated proteins recorded at 5 (grey line, shaded area), 10, 50 and 100 (black line) mV s^{-1}. Data recorded in 50 mM HEPES, 50 mM NaCl, pH 7 at 4 °C.

(Left) Scan rate dependence of voltammetric features.

Upper part of panels. Black: total moles of electrons (Q_{tot}) giving rise to the oxidative (circles) and reductive (triangles) peaks normalized to those at 10 mV s^{-1} (black). Red: ratio of mol. electrons in the low potential peak (Q_{hi}) to mol. electrons in the high potential peak (Q_{lo}) for oxidative (circles) and reductive (triangles) peaks.

Lower part of panels. Red: peak potentials (E_p) for oxidative (circles) and reductive (triangles) peaks at low (blue) and high (red) potential. Heterogeneous rate constants (k_o / s^{-1}) were obtained from fits to the data using Jellyfit v2.1 available from (https://www.fbs.leeds.ac.uk/jeukengroup/freeware/Jellyfit.php). k_0 values used for low/high E_m peaks were 1.06/1.48 s^{-1} (*Cj* WT), 0.78/0.67 s^{-1} (*Cj* C138M) and

0.87/1.38 s^{-1} (*Cj* C138H). E_m was equal to the average of the corresponding E_p values. Examples of similar analyses can be found in Jeuken, L.J.C., McEvoy, J.P. & Armstrong, F.A. (2002) J. Phys. Chem. B 106 2304-2313.

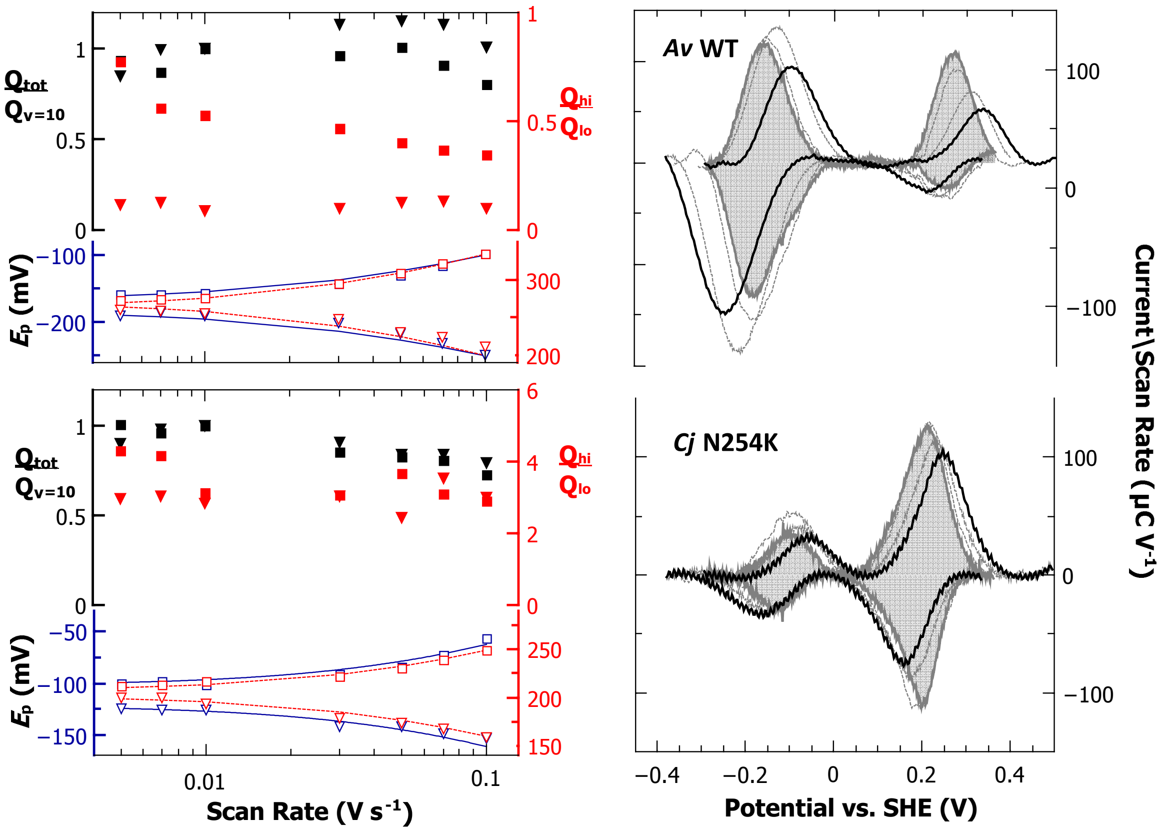

Figure S4. Scan-rate dependence of cyclic voltammetry for *Av*TsdA and *Cj*TsdA N254K adsorbed on IO-ITO electrodes.

(Right) Representative baseline subtracted cyclic voltammograms for the indicated proteins recorded at 5 (grey line, shaded area), 10, 50 and 100 (black line) mV s^{-1}. Data recorded in 50 mM HEPES, 50 mM NaCl, pH 7 at 4 °C.

(Left) Scan rate dependence of voltammetric features.

Upper part of panels. Black: total moles of electrons (Q_{tot}) giving rise to the oxidative (circles) and reductive (triangles) peaks normalized to those 10 mV s^{-1} (black). Red: ratio of mol. electrons in the high potential peak (Q_{hi}) to mol. electrons in the low potential peak (Q_{lo}) for oxidative (circles) and reductive (triangles) peaks.

Lower part of panels. Red: peak potentials (E_p) for oxidative (circles) and reductive (triangles) peaks at low (blue) and high (red) potential. Heterogeneous rate constants (k_o / s^{-1}) were obtained from fits to the data using Jellyfit v2.1 available from (https://www.fbs.leeds.ac.uk/jeukengroup/freeware/Jellyfit.php). k_0 values used for low/high E_m peaks were 0.67/0.72 s^{-1} (*Av* WT) and 1.32/1.22 s^{-1} (*Cj* N254K). E_m was equal to the average of the corresponding E_p values. Examples of similar analyses can be found in Jeuken, L.J.C., McEvoy, J.P. & Armstrong, F.A. (2002) J. Phys. Chem. B 106 2304-2313.

Table S1. Peak wavelengths (λ) and intensities (Δε) for low-spin Fe(III) charge-transfer transitions in nIR MCD. ox = oxidized form, asc = ascorbate reduced form.

λ / nm<br>(Δε / M^{-1} cm^{-1} T^{-1})		Heme 1			Heme 2	
		His/Cys^{-}	His/His	His/Met	His/Lys	His/Met
*Av*TsdA<br>(407/208)*	ox				1505 (1.6)	
	asc	1240<br>(0.42)				
*Cj*TsdA<br>(415/190)	ox	1215				1825<br>(0.74)
	asc	1225<br>(0.50)				
*Cj*TsdA C138M<br>(413/210)	ox			1735†<br>(0.51)		1825‡<br>(0.74)
	asc					
*Cj*TsdA C138H<br>(411/230)	ox		1480			1820<br>(0.82)
	asc		1480<br>(1.03)			
*Cj*TsdA N254K<br>(408/200)	ox	1250			1525 nm<br>(0.75)	1825<br>(0.47)
	asc	1250<br>(0.41)				

* The Soret absorption peak wavelength and extinction coefficients (nm/$mM^{-1}cm^{-1}$) are shown in brackets for each species.

† by subtraction.

‡ assumed to be the same as *Cj*TsdA.

Table S2. E_m values derived from modelling of voltammograms, see Methods for details.

	Heme 1		Heme 2			
*Av*TsdA	His/Cys^{-}	−181 mV	His/Lys	−129 mV	His/Met	+266 mV
*Cj*TsdA	His/Cys^{-}	−186 mV			His/Met	+172 mV
*Cj*TsdA C138M	His/Met	+94 mV			His/Met	+174 mV
*Cj*TsdA C138H	His/His	−100 mV			His/Met	+160 mV
*Cj*TsdA N254K	His/Cys^{-}	−115 mV	His/Lys	−171 mV	His/Met	+203 mV

Table S3. Moles of electrons transferred (*Q*) in the baseline-subtracted and modelled peak areas for cyclic voltammetry of adsorbed TsdA proteins. Bolded values (± σ) are average of three scans performed at 10 mV s^{-1} in 50 mM HEPES, 50 mM NaCl, pH 7, 4 °C with the exception of *Cj*TsdA

N254K scan 1. Italicized values are for the modelled contributions shown in Fig 5 and 8 with grey values assigned to Heme 1 and blue values assigned to Heme 2. Ox = oxidized, red = reduced, lo = low potential, hi = high potential.

	**Low Potential Peak (mol *e*⁻)**		**High Potential Peak (pmol *e*⁻)**		**Total (pmol *e*⁻)**	
**Enzyme**	Q^{ox}_{lo}	Q^{red}_{lo}	Q^{ox}_{hi}	Q^{red}_{hi}	Q^{ox}_{tot}	Q^{red}_{tot}
***Cj*TsdA**	**41 (± 10)**<br>*31*	**52 (± 10)**<br>*41*	**166 (± 10)**<br>*145*	**135 (± 21)**<br>*124*	**207 (± 10)**<br>*176*	**187 (± 21)**<br>*165*
***Av*TsdA**	**176 (± 10)**<br>*114, 31*	**269 (± 21)**<br>*135, 83*	**93 (± 10)**<br>*93*	**21 (± 10)**<br>*31*	**269 (± 10)**<br>*238*	**300 (± 21)**<br>*249*
***Cj*TsdA C138M**			**373 (± 21)**<br>*176, 176*	**352 (± 10)**<br>*166, 145*	**373 (± 21)**<br>*352*	**352 (± 10)**<br>*311*
***Cj*TsdA C138H**	**155 (± 10)**<br>*135*	**124 (± 10)**<br>*104*	**207 (± 10)**<br>*218*	**238 (± 21)**<br>*218*	**373 (± 10)**<br>*353*	**352 (± 21)**<br>*322*
***Cj*TsdA N254K scan 1**	**52**<br>*21, 41*	**145**<br>*62, 73*	**165**<br>*124*	**83**<br>*52*	**218**<br>*187*	**228**<br>*187*
**steady-state**	**52 (± 10)**<br>*52*	**31 (± 10)**<br>*41*	**155 (± 10)**<br>*124*	**135 (± 21)**<br>*104*	**207 (± 10)**<br>*176*	**166 (± 10)**<br>*145*
